# Retinal Dysfunction in Hypertensive Patients with Atherosclerotic Plaque Detected by Carotid Doppler Ultrasound: An Optical Coherence Tomography Angiography Assessment

**DOI:** 10.3390/life16030436

**Published:** 2026-03-09

**Authors:** Irina Barca, Vasile Potop, Stefan Sorin Arama

**Affiliations:** 1 Department of Physiology, “Carol Davila” University of Medicine and Pharmacy, 050474 Bucharest, Romania; sorin.arama@umfcd.ro; 2 Department of Ophthalmology, “Carol Davila” University of Medicine and Pharmacy, 050474 Bucharest, Romania

**Keywords:** hypertensive retinopathy, atherosclerotic plaque, optical coherence tomography angiography, carotid Doppler ultrasonography, retinal vasculature

## Abstract

**Background**: Our study aimed to evaluate whether OCTA can detect retinal dysfunction in hypertensive patients with atherosclerotic plaque in order to improve early detection of vascular changes and to better adjust treatment protocols. Therefore, we can potentially reduce the rate of ocular, cardiovascular and cerebral complications of hypertension and of dyslipidemia. **Methods**: We performed a study on hypertensive patients with dyslipidemia undergoing specific treatment. Ten OCTA parameters, the presence of carotid plaque on carotid Doppler ultrasound and three types of antihypertensive drugs were analyzed. An increased carotid intima-media thickness (IMT) (≥1.0 mm) or the presence of carotid plaque was defined as subclinical atherosclerosis. We correlated classes of medication with OCTA parameters and with Doppler assessment. **Results**: In the final study, we included 196 eyes of 98 patients; 51 subjects had carotid plaques. Three groups were formed: antihypertensive monotherapy, including Angiotensin-converting enzyme inhibitor (ACEI) or Calcium channel blocker (CCB) + statins, and combined antihypertensive therapy, including ACEI/Angiotensin Receptor Blocker (ARB) + statins. We found statistically significant results in the presence of atherosclerotic plaques as follows: increased avascular zone (FAZ) and decreased vascular flow area (VFA) in the ACEI group, increased FAZ Circularity and a reduction in Density Total in the CCB lot, higher values of non-flow area (NFA), FAZ Area and decreased Density Total in the ACEI/ARB group. **Conclusions**: The strongest correlations we found were between increased hypertension, decreased retinal microcirculation and the presence of atherosclerotic plaques in patients using combined antihypertensive therapy and statins. The results indicate that subjects with multiple therapies, advancing hypertensive retinopathy and atherosclerotic carotid plaques display a deficit in retinal vascularization. OCTA can provide early detection of microvascular changes in hypertension associated with dyslipidemia and carotid plaques. Thus, by correlating OCTA and carotid Doppler ultrasound, antihypertensive and statin therapy can be adjusted and disease risk stratification can be obtained.

## 1. Introduction

Hypertension represents a global disease burden [1]. Due to aging populations, the number of patients continues to increase. The optimal control of blood pressure is an important target for improving public health [2]. Systemic disease complications, especially cardiovascular complications, are the main causes of mortality [3,4].

Hypertension is associated with multiple retinal microvascular changes, such as generalized and focal arteriolar narrowing, arterio-venous nicking, and narrowed and decreased arterioles [5,6,7,8]. Previous articles have reported partial regression of these changes with antihypertensive treatment [9,10], but different antihypertensive regimens have not been compared in large studies.

Around 44% of the studied patients had been diagnosed with hypertensive retinopathy [11], presenting a high risk of cardiovascular disease mortality [12,13]. Changes in retinal microcirculation are associated with hypertension and predict cardiovascular mortality [8]. Thus, it has been suggested that hypertensive retinopathy could be a valuable indicator of severe hypertension complications [14] requiring more aggressive antihypertensive treatments [4,15]. Therefore, frequent ophthalmological examinations and therapy adjustments are essential in patients with chronic disease.

A number of researchers have considered the impact of different stages of hypertensive retinopathy on optical coherence tomography angiography (OCTA) examination [4,16]. The antihypertensive treatment has been proven to have an influence on retinal microcirculation [8]. Previous studies did not consider the effect of medication on vessel density in patients with hypertension and dyslipidemia [16,17,18,19].

OCTA may reveal microvascular abnormalities before they become clinically apparent, thus proving to be a more accurate evaluation of the microvasculature of the retina than other types of investigation [20,21]. It has been demonstrated that the risk of cardiovascular disease can be monitored by quantifying retinal microvascular alterations using OCTA [21,22,23,24].

OCTA can quantify and assess vessel caliber, tortuosity, capillary perfusion and foveal vascular zone characteristics. These parameters have been associated with coronary heart disease, stroke and heart failure [25]. Vessel density loss and foveal avascular zone alterations occur in hypertension, coronary disease, heart failure, and stroke. Arteriolar narrowing and venular widening predict incident coronary heart disease. A reduction in retinal microcirculation can be associated with increased major adverse cardiovascular events [26].

Treatment strategies for hypertensive patients include combined drugs or monotherapy. The major classes of antihypertensive drugs are as follows: Angiotensin-converting enzyme inhibitors ((ACEIs), blocking the production of angiotensin that narrows blood vessels), Calcium channel blockers ((CCBs), relaxing blood vessels by preventing calcium from entering heart and artery cells), Angiotensin II Receptor Blockers ((ARBs), blocking the action of angiotensin), beta-blockers (slowing the heart rate and reducing the force of its contractions), alpha-blockers (relaxing muscles in blood vessels to improve blood flow), diuretics (eliminating sodium and water, reducing blood volume) and vasodilators (directly relaxing blood vessels).

The imaging of carotid plaques and the measurement of the internal carotid artery stenosis degree represent a simple, cost-effective method, and the reported overall accuracy is up to 92% [20,27]. A significant percentage of patients present with subclinical atherosclerosis on Doppler ultrasound [28,29,30,31,32]. Coronary artery calcification (measured by computed tomography and carotid intima-media thickness) and plaque assessment using B-mode ultrasound can identify subclinical atherosclerosis [30]. Atherosclerotic plaque detection is therefore non-invasive and rapid, and it provides cardiovascular disease risk stratification.

However, not only the presence of carotid plaque leads to atherosclerosis, but also an increased carotid intima-media thickness (IMT) (≥1.0 mm) is a predictor of future atherosclerotic cardiovascular diseases [33,34]. Both parameters can be easily determined on carotid Doppler ultrasound. A higher value of IMT or the presence of carotid plaque was defined as subclinical atherosclerosis.

Our research aimed to quantify and compare retinal microvasculature changes found through OCTA in patients with hypertensive retinopathy, dyslipidemia and atherosclerotic plaque. Our subjects were undergoing treatments with three different types of antihypertensives (ACEI, CCB, ACEI/ARB) and statins, both combined and statin monotherapy.

The purpose of our research was to discover retinal vasculature dysfunction, such as larger areas of reduced retinal vascular flow, reduced vascular density and skeleton, in patients with atherosclerotic plaques, hypertensive retinopathy and dyslipidemia.

We aimed to investigate early detection of vascular changes and to use the data provided by OCTA in order to better adjust treatment protocols.

## 2. Materials and Methods

### 2.1. Study Population

Our research was conducted according to the tenets of the Declaration of Helsinki, and informed consent was obtained from all patients. The study was approved by the Ethics Committee of the “Regina Maria: The Healthcare Network” (645 bis/14.12.2022).

This prospective cohort study was performed in Eastern Europe, specifically Romania, including ninety-eight patients with systemic hypertension, dyslipidemia and atherosclerotic plaques. Nine patients were excluded due to poor-quality images of the OCTA scans. All participants underwent comprehensive ophthalmologic examination, carotid Doppler ultrasonography and blood testing.

For the purpose of this study, we selected patients with chronic hypertension, chronic hypertensive retinopathy and secondary dyslipidemia. The treatments for hypertension and dyslipidemia were evaluated in order to study the correlation between retinal microvasculature and atherosclerotic disease.

Using fundoscopy, we included in our research subjects with hypertensive retinopathy according to the Keith–Wagener–Barker and Mitchell–Wong classification systems [35]. Based on carotid Doppler ultrasound evaluation, we determined the presence of atherosclerotic plaques.

Subjects with the following ophthalmological diseases were excluded with the help of fundoscopy, retina and optic nerve OCT: glaucoma, age-related macular degeneration, central serous retinopathy, pigment epithelial detachments, diabetic retinopathy, macular telangiectasia, epiretinal membranes, cystoid macula edema, vitreomacular interface disorders and other pathologies affecting retinal vasculature. Internal carotid artery stenosis (ICAS) related complications, such as ocular ischemic syndrome, central or branch retinal artery occlusion and anterior ischemic optic neuropathy, were not part of this study.

Patients with systemic pathologies were also excluded from our study: heart failure, aortic stenosis or aneurysm, any other obstruction of the proximal vascular system from the internal carotid artery (ICA), hematologic diseases, vasculitis, autoimmune diseases, cerebrovascular accidents, neurodegenerative diseases and malignancies.

We performed measurements according to protocol: blood testing, ophthalmological examinations and carotid Doppler ultrasound every three months, and blood pressure was measured twice per month.

According to the American Heart Association criteria, hypertension is defined as systolic blood pressure (SBP) ≥ 130 mm Hg and diastolic blood pressure (DBP) ≥ 80 mm Hg [16,36,37,38]. A blood pressure higher than 180/120 mm Hg is considered a hypertensive emergency, and our study excluded this pathology. Primary (essential) hypertension was studied in this current research. Hypertension is a progressive, chronic condition, and a longer duration of disease significantly increases the risks of cardiovascular disease events, stroke, and mortality, according to a 2022 study published by Yan Zheng et al. [39].

Our research included hypertensive patients with a disease duration between 5 and 10 years (11 subjects), 10 and 15 years (34 participants) and more than 15 years (53 subjects). Thus, the majority of our patients had an increased risk of complications and mortality. During the time we conducted our study, no hypertension-related events were diagnosed.

Participants with secondary dyslipidemia were included in this research. Hypercholesterolemia (high LDL levels), hypertriglyceridemia (high triglyceride levels) and combined hyperlipidemia (raised levels of both LDL and triglycerides) were analyzed. We did not investigate alpha lipoproteinemia.

Smoking habit was assessed with the single-categorized question “Are you currently a smoker?” Blood tests were performed every three months, including: total cholesterol, high-density lipoprotein (HDL), low-density lipoprotein (LDL) and triglycerides. Age, sex and environment were also noted.

Each subject underwent a complete ophthalmic examination, including best-corrected visual acuity, refraction, slit-lamp examination, measurement of the intraocular pressure, fundoscopy and OCTA.

Optopol Revo NX 130 OCTA of the macula and carotid Doppler ultrasonography using G.E. Vivid T8 Cardiovascular Ultrasound were performed on all patients every three months. OCTA evaluated the foveal avascular zone (FAZ), the non-flow area (NFA), the vascular flow area (VFA), the retinal vascular density and perfusion.

OCTA can provide an assessment of the following eye layers: retina, vitreous, Superficial Capillary Plexus (SCP), Superficial Vascular Plexus (SVC), Radial Peripapillary Capillary Plexus (RPCP), Deep Capillary Plexus, Deep Vascular Plexus (DVC), Intermediate Capillary Plexus (ICP), outer retina layers, choriocapillaris, Choroidal Vessels, and Depth Coded.

On OCTA, the FAZ is primarily measured at the SCP. OCTA devices also allow for measurement of the FAZ in the DVC, as both layers are often analyzed. The NFA is measured to quantify capillary non-perfusion or ischemia in the SCP and the DVC. The VFA is specifically evaluated in the choriocapillaris layer. The capillary density and skeleton density are primarily measured in the superficial retinal layer and the deep retinal layer.

Carotid Doppler ultrasonography using G.E. Vivid T8 Cardiovascular Ultrasound determined the presence or absence of atherosclerotic plaques.

Eyes with poor-quality images or signal strength, with significant artifacts that obscured the vascular area of interest, with high myopia and dense cataracts, were excluded as visualization of the retina was impaired.

Patients were divided into three groups according to the type of medication given by their cardiologist: antihypertensive monotherapy (ACEI or CCB) + statins and combined antihypertensive therapy (ACEI/ARB) + statins.

### 2.2. Examination and OCTA Analysis

OCTA was performed on the macula using Angio-OCT Optopol Revo NX 130 with AI technology, thus improving the tomogram quality and the visualization of OCTA data.

For the purpose of our research, we performed a 6.0 × 6.0 mm^2^ macula HD scan, centered on the fovea of both eyes. The measured areas were: total, superior, inferior, center, inner, superior inner, inferior inner, outer, superior outer and inferior outer. The Early Treatment of Diabetic Retinopathy Study (ETDRS) grid was also calculated.

The software analyzed the superficial layer (offset of the inner limiting membrane (ILM)—0 µm; inner plexiform layer/inner nuclear layer (IPL/INL)—15 µm), the deep layer (offset of the IPL/INL—15 µm; IPL/INL—70 µm) and the choriocapillaris (offset of Bruch’s membrane (BM): top BM—30 µm; bottom BM—45 µm).

OCTA provided an assessment of the FAZ, NFA, and VFA, the retinal vascular density and the skeleton. The FAZ, NFA and VFA were detected and measured using the semi-auto area tool of the software. Manual measurements were performed in six scans, where the semi-automated measurement was inaccurate. Density and skeleton displays detect abnormal retinal vasculature and can be used to obtain repeatable quantitative results.

### 2.3. Statistical Analysis

All analyses were performed using IBM Statistical Package for the Social Sciences (SPSS) V26, JASP V0.95.4 and Microsoft Office Professional Plus 2021.

The whole sample of 196 eyes from 98 subjects was divided into 3 groups regarding medication: the antihypertension monotherapy groups, with 34 (Angiotensin-converting enzyme inhibitor (ACEI) drugs) and 30 subjects (Calcium channel blocker (CCB) medication), and the combined antihypertension therapy group (ACEI and Angiotensin Receptor Blockers (ARBs)), with 34 patients.

Using descriptive statistics, the following indicators were calculated: mean and standard deviation. We also analyzed age, sex and smoking status.

An Analysis of Variance (ANOVA) was performed in order to compare the means of the three lots and to determine if at least one is significantly different from the others.

The Brown–Forsythe test was used to check for homogeneity of variances across the groups.

The normality of the distribution was calculated using the Shapiro–Wilk test for multivariate normality.

The Kruskal–Wallis test determined if there were statistically significant differences between lots. To compare different therapy groups, we also used Dunn’s post hoc test.

Spearman and Pearson correlations were used among clinical values, OCTA parameters, and carotid Doppler analysis of atherosclerotic plaques.

After the ANOVA analysis, we performed post hoc tests with Bonferroni corrections, making multiple pairwise comparisons between group means.

## 3. Results

### 3.1. Descriptive Analysis

A total of 98 subjects and 196 eyes with hypertensive retinopathy and dyslipidemia were enrolled in our study.

Males (55.1%) and non-smokers (75.5%) represented the majority when determining demographics in all three groups. No statistical differences were found between groups when calculating age, BMI and area. The descriptive analysis is displayed in Table 1.

Our research enrolled patients with antihypertensive monotherapy, either ACEI or CCB, and combined antihypertensive therapy (ACEI/ARB). All subjects were taking statins, both monotherapy and combined statin therapy. We did not form other smaller groups by splitting the last two types of drugs, in order not to lose statistical power. Three groups were formed: an antihypertensive monotherapy (ACEI) + statins group, with 34 patients, an antihypertensive monotherapy (CCB) + statins group, with 30 subjects, and a combined antihypertensive therapy (ACEI/ARB) + statins group, with 34 participants.

With increasing BMI values and age, the risk of developing atherosclerosis is higher [40]. Thus, the most statistically significant correlations among demographic parameters and ultrasound were calculated. Pearson and Spearman tests were performed. The correlation among BMI and ultrasound parameters proved to be the most statistically significant. An increased age was linked to higher levels of ICA end-diastolic velocity (EDV) and common carotid artery (CCA) IR. Smokers displayed lower values of external carotid artery (ECA) peak systolic velocity (PSV). The results can be found in the Supplementary Materials.

The presence of increased carotid intima-media thickness (IMT) above the normal limit (>1.0 mm) or the presence of carotid plaque was defined as subclinical atherosclerosis. Overall, 51 subjects (52.04%) were confirmed to have atherosclerotic plaque on carotid Doppler ultrasound, and five participants had an IMT ≥ 1.0 mm (5.1%).

### 3.2. Analysis of OCTA Parameters in Patients with Atherosclerotic Plaque

OCTA analyses different retinal layers (superficial and deep retinal vasculature) and choriocapillaris; thus, the machine provides specific quantitative measurements such as: FAZ, NFA, VFA, density and skeleton.

The Shapiro–Wilk test for multivariate normality in all antihypertensive therapies was calculated for the following groups: ACEI + statins (SW-value = 0.932, *p* < 0.001), CCB (SW-value = 0.944, *p* < 0.001) and ACEI/ARB (SW-value = 0.923, *p* < 0.001).

An ANOVA test was used in order to compare the means of the three medication groups and to investigate if at least one group mean is significantly different from the others. The results are shown in Table 2.

The ANOVA test determined statistical differences between the three medication groups for NFA Area, FAZ Area, FAZ Circularity, VFA Flow Area and density (Total and EDTRS) (*p* < 0.05). Thus, post hoc tests were performed next.

The post hoc test with Bonferroni corrections was performed after the ANOVA test, making multiple pairwise comparisons between group means. Table 3 shows the obtained data. Statistically significant results (*p* < 0.05) were found for: NFA Area and FAZ Area (when comparing the combined with antihypertensive monotherapy + statins group) and FAZ Circularity, VFA Flow Area, and Density Total and ETDRS (when comparing antihypertensive monotherapy + statins with ACEI/ARB + statins).

The Kruskal–Wallis test was performed to check for statistically significant differences between lots. Therefore, six OCTA parameters were found when analyzing all therapy groups. The most relevant results obtained were differences between treatment groups for NFA Area, FAZ Area, VFA Flow Area and Density (Total and ETDRS). Table S8 in the Supplementary Materials shows the Kruskal–Wallis test.

Dunn’s post hoc comparisons were performed in order to evaluate the ACEI/ARB + statins, the ACEI + statins and the CCB + statins groups. The results are displayed in the Supplementary Materials in Table S9. Statistically significant results (*p* < 0.05) were found for: NFA Area, FAZ Area, FAZ Circularity, VFA Flow Area and density (Total and ETDRS).

A multivariate analysis with MANCOVA was performed next.

Box’s Test of Equality of Covariance Matrices was statistically significant (Box M = 455,876, F = 3,517, *p* < 0.001), indicating a lack of homogeneity of data. Therefore, a Pillai’s Trace test was used.

Multivariate tests are displayed in Table 4. The results indicated a statistically significant multivariate effect of medication on the combined OCTA parameters after adjusting for covariates (*p* < 0.001). Both Pillai’s Trace = 0.564, F (20,160) = 3.139, *p* < 0.001, and partial η^2^ = 0.282, and Wilks’ Lambda = 0.512, F (20,158) = 3.139, *p* < 0.001, and partial η^2^ = 0.284, showed a large effect size.

Among the covariates, total cholesterol indicated a significant multivariate effect on OCTA parameters (Pillai’s Trace = 0.230, F (10.79) = 2.357, *p* = 0.017, partial η^2^ = 0.230). Wilks’ Lambda = 0.770, F (10.79) = 2.357, *p* < 0.001, and partial η^2^ = 0.230 resulted in a large effect size. Other covariates, including SBP, DBP, HDL, LDL, triglycerides and disease duration, did not show significant multivariate effects (*p* > 0.05).

Tests of Between-Subjects Effects are displayed in Table 5, which shows the effects of independent variables and covariates on OCTA parameters. The medication group had a significant statistical effect (*p* < 0.05) on the following OCTA parameters: NFA Area, FAZ Area, FAZ Circularity, VFA Flow Area and Skeleton ETDRS. Partial Eta squared displayed a large effect size of FAZ Area. When analyzing medication groups and skeleton, a borderline value of *p* = 0.053 resulted. Clinical parameters, including blood pressure and lipids, had a statistically significant effect on OCTA parameters (*p* < 0.05).

Estimated Marginal Means and pairwise comparisons showed the adjusted means for the medication groups for each OCTA parameter, after eliminating the influence of the covariates. Small differences were found between means. The results are included in the Supplementary Materials in Table S11.

Table 6 displays the results of pairwise comparisons. Only post hoc comparisons are highlighted. A statistically significant difference between groups for each OCTA parameter was obtained following Bonferroni correction and adjusted means. *p*-values for VFA Flow and FAZ Circularity were borderline.

Spearman and Pearson correlations were used among OCTA parameters, carotid Doppler ultrasound and clinical measurements. The statistically significant results obtained are displayed in Table 7, Table 8 and Table 9 for the three treatment groups. All other correlations can be found in the Supplementary Materials.

In the ACEI + statins lot, the performed tests showed that increased values of DBP and SBP are correlated with a larger area of decreased blood flow or FAZ and with low density and skeleton. Total cholesterol was significantly correlated with NFA Area (r = 0.477; *p* = 0.004), (rho = 0.541; *p* = 0.001). A significant effect was obtained when correlating the presence of carotid plaque with FAZ Area (r = 0.540; *p* ≤ 0.001), (rho = 0.526; *p* = 0.001), and with VFA Area (r = −0.409; *p* = 0.016), (rho = −0.430; *p* = 0.011). Thus, a retinal dysfunction with poor retinal vascularization can be suspected.

When analyzing the CCB + statins group, the results showed that higher blood pressure and raised levels of LDL and HDL can be linked to extensive non-flow areas, larger avascular areas and decreased capillary perfusion. Total cholesterol was significantly correlated only with SPB (r = 0.379; *p* = 0.039). The presence of atherosclerotic plaques proved to be most significant when linked to FAZ Circularity (r = −0.406; *p* = 0.026), (rho = −0.409; *p* = 0.025) and Density Total (r = 0.400; *p* = 0.028), (rho = 0.389; *p* = 0.034).

In the ACEI/ARB + statins group, the strongest correlations we found were among clinical values (blood pressure with LDL and HDL) and among OCTA parameters: FAZ (Area and Perimeter) with density and skeleton. Atherosclerotic carotid plaques were correlated with the three OCTA parameters; thus, significant results were obtained: NFA (r = −0.558; *p* ≤ 0.001), (rho = −0.555; *p* ≤ 0.001), FAZ Area (r = 0.405; *p* = 0.017), (rho = 0.394; *p* = 0.021) and Density Total (r = −0.527; *p* = 0.001), (rho = −0.509; *p* = 0.002).

After performing calculations for all therapy lots, statistically significant results showed that increased SPB, DBP, total cholesterol and LDL are correlated with decreased VFA, higher values of avascular and non-flow areas. Therefore, subjects with combined antihypertensive therapy and statins displayed increasing blood pressure, dyslipidemia and progressive retinopathy. Among the strongest correlations we obtained, atherosclerotic plaques were linked to NFA Area (r = −0.242; *p* = 0.017), (rho = −0.237; *p* = 0.019), FAZ Area (r = 0.222; *p* = 0.028), (rho = 0.214; *p* = 0.034) and Density Total (r = −0.274; *p* = 0.006), (rho = −0.219; *p* = 0.030). Thus, poor retinal perfusion, such as decreased capillary density, increased non-flow and avascular areas, is correlated with the presence of atherosclerotic plaques.

## 4. Discussion

OCTA was used in a number of research studies in order to study systemic cardiovascular disease.

An increasing number of articles have indicated that retinal microvascular abnormalities may be used as a potential indicator for microvascular, cardiovascular and neurodegenerative diseases [21,41,42].

One of the newly published papers in December 2025 belongs to the team of author Ting Wang [26]. Retinal microvascular biomarkers for cardiovascular risk stratification, OCTA and AI were studied. According to the research team, AI models trained on retinal images, integrated with clinical variables, achieve discrimination comparable to or exceeding traditional risk scores. Their conclusion was that retinal microvascular imaging is a non-invasive approach to cardiovascular risk stratification. Their review showed that fundus-based measures, such as arteriolar narrowing, venular widening, tortuosity, and the severity of diabetic retinopathy, are consistently associated with future coronary heart disease, stroke, heart failure, and death. Our current study examines another retinal vascular disease, hypertensive retinopathy, not diabetic. But both studies apply OCTA when studying retinal microcirculation, correlate ocular and systemic disease, and evaluate OCTA parameters. Two of our other studies were similar to the article published in 2025. One of two investigated progression in hypertensive patients with dyslipidemia using OCTA and AI [43], and the other studied the relevance of OCTA in screening hypertensive subjects with carotid artery stenosis [23].

In 2020, J. Yoon et al. investigated the associations between macular microvasculature assessed by OCTA and subclinical atherosclerosis in patients with type 2 diabetes [33].

Patients received evaluations such as carotid ultrasonography and OCTA. The presence of increased IMT > 1.0 mm or carotid plaque was defined as subclinical atherosclerosis. OCTA characteristics focused on the FAZ and parafoveal vessel density. The decrease in density around the FAZ and the loss of density in the parafoveal area of both superficial (SVP) and Deep Capillary Plexus (DVP) were significantly associated with subclinical atherosclerosis, suggesting that common pathogenic mechanisms might predispose to diabetic micro- and macrovascular complications. We found similarities in our research: with the use of OCTA and Doppler ultrasound and the evaluation of IMT, the results showed that the FAZ was increased while the density was decreased in the presence of carotid plaques. We also found differences: our subjects were hypertensive, not diabetic, and only five out of 98 patients had an IMT above 1 mm. Subclinical atherosclerosis was present in both studies.

The detection of subclinical carotid artery changes while patients are asymptomatic represents an important public health and economic goal [28]. OCTA may be a valuable tool in detecting early microvascular alterations in the retina, even before they become clinically apparent. Retinal circulation may also be used as an indicator of cardiovascular diseases [44,45].

It is important for doctors to be familiar with ophthalmic symptoms and signs caused by carotid atherosclerosis in order to provide an early diagnosis and to take appropriate measures, like adjusting treatment strategies, in order to prevent a stroke.

OCTA can help identify early changes in retinal microcirculation; therefore, it could provide cardiologists with more information when choosing different treatment strategies for hypertensive patients with dyslipidemia and carotid plaques.

Our research found that the presence of atherosclerotic plaques is linked to larger FAZ measurements in all three lots, with decreased total density in the CCB and ACEI/ARB groups, reduced VFA in the ACEI therapy lot and higher NFA values in the combined antihypertensive group. The most statistically significant OCTA parameters proved to be FAZ Area and Density Total.

The most recent paper published in 2026 by X. Du, J. Dong and Y. Li explores the development of an OCTA-based risk prediction model for retinopathy in patients with obstructive sleep apnea and hypertension [46]. Their objective was to identify risk factors for retinopathy in patients with obstructive sleep apnea and hypertension and to develop a machine learning-based risk prediction model by integrating OCTA parameters. The study enrolled 98 hypertensive patients, the same number of subjects as our research. Compared with the non-retinopathy group, the retinopathy group exhibited significantly elevated apnea–hypopnea index, oxygen desaturation index and impaired OCTA retinal vascularization. Their research concluded that by integrating OCTA biomarkers among others, a more precise risk stratification for retinopathy in obstructive sleep apnea–hypertension can be obtained. The paper proved a link between microvascular dysfunction and nocturnal hypoxemia; thus, a quantifiable tool for early risk prediction of target organ damage can be found. Its clinical translation potential lies in guiding personalized interventions in order to reduce systemic complications in high-risk populations. Similar to their work, our study demonstrated that OCTA can be used as a tool to detect retinal dysfunction and alterations in retinal circulation. One of our goals was similar to theirs: to help identify the proper antihypertensive medications for each group.

We would like to acknowledge several limitations of our study.

First, the sample size consisted of 196 eyes of 98 subjects. Only five subjects had both carotid plaques and an IMT ≥ 1 mm. A larger number of participants is needed in order to provide more insight into both Doppler measurements.

Second, our research included only hypertensive retinopathy stages I and II, as we did not find patients with stages III and IV of the disease. We did not determine Lipoprotein A and B, and these tests could possibly provide more accurate data.

Third, no analysis was performed regarding other types of antihypertensive medication usually prescribed to hypertensive patients. This would include combined medication or monotherapy, such as ARBs, beta-blockers, diuretics, alpha-blockers, vasodilators, and drugs, that could have had a potential effect on OCTA results. Also, we did not investigate the type of statins in combined or monotherapy.

The possible association between blood pressure control, dyslipidemia and OCTA vascular changes in hypertensive patients should be investigated further.

## 5. Conclusions

The strongest correlations we found were between increased hypertension, presence of atherosclerotic plaques and decreased retinal microcirculation in patients using combined antihypertensive therapy and statins.

We found statistically significant results in the presence of atherosclerotic plaques as follows: in the ACEI group, increased FAZ Area and decreased VFA Area; in the CCB lot, a reduction in Density Total and increased FAZ Circularity; and in the ACEI/ARB group, higher NFA and FAZ Area and decreased Density Total.

Patients with combined antihypertensive therapy and statins displayed increasing blood pressure, dyslipidemia and progressive retinopathy. The strongest correlations we found were between atherosclerotic plaques and NFA Area, FAZ Area and Density Total. Therefore, poor retinal perfusion, such as decreased capillary density, increased non-flow and avascular areas, is present in patients with atherosclerotic plaques.

The results indicate that subjects with multiple therapies, with a poor control of dyslipidemia, raised blood pressure, advancing hypertensive retinopathy and atherosclerotic carotid plaques, display a deficit in retinal vascularization.

OCTA can provide early detection of microvascular changes in hypertension associated with dyslipidemia and carotid plaques. In patients with combined therapies and raised blood pressure, OCTA detected larger avascular and non-flow areas and reduced vascular density and skeleton.

Thus, by correlating data obtained with the help of OCTA and carotid Doppler ultrasound, antihypertensive and statin therapy can be adjusted. Furthermore, disease risk stratification and personalized interventions, in order to reduce ocular and systemic complications in high-risk populations, can be obtained.

Further studies with larger patient groups, additional carotid Doppler ultrasound parameters and medication use could provide more information on this matter.

Often, the only manifestations in patients with carotid atherosclerotic disease are ophthalmologic symptoms and signs. Therefore, a complete ocular examination, including OCTA examinations, complementary to carotid Doppler imaging and other specific investigations, could easily identify patients at risk of ischemic stroke and could refer them for early intervention or a change in medication.

## Figures and Tables

**Table 1:** Demographic parameters. BMI: body mass index, ACEI: Angiotensin-converting enzyme inhibitor; CCB: Calcium channel blocker; ARB: Angiotensin II Receptor Blocker. * χ^2^ test for gender, smoking habit and area. Kruskal–Wallis test for age and BMI. ** Age and BMI calculated as M ± SD. M: mean; SD: standard deviation; n: number of subjects per group.

Demographic Parameters	Medication Groups			*p*-Value *
	ACEI + Statins	CCB + Statins	ACEI/ARB + Statins	
	(N = 34)	(N = 30)	(N = 34)	
**Gender**				0.504
Female	18 (53.0%)	12 (40.0.8%)	14 (41.2%)	
Male	16 (47.1%)	18 (60.0.2%)	20 (58.8%)	
**Smoker?**				0.096
YES	4 (19.1%)	10 (33.3%)	10 (29.4%)	
NO	30 (80.9%)	20 (63.2%)	24 (70.6%)	
**Area**				0.278
Rural	18 (52.9%)	10 (33.3%)	14 (41.2%)	
Urban	16 (47.1%)	20 (66.7%)	20 (58.8%)	
**Age** ** (years)	59.9 ± 9.1	55.7 ± 9.4	54.2 ± 11.6	0.137
**BMI** ** (kg/m^2^)	28.3 ± 3.6	27.7 ± 2.8	28.2 ± 3.5	0.680

**Table 2:** ANOVA test. OCTA: optical coherence tomography angiography; FAZ: foveal avascular zone; NFA: non-flow area; VFA: vascular flow area; ETDRS: Early Treatment of Diabetic Retinopathy Study; ACEI: Angiotensin-converting enzyme inhibitor; CCB: Calcium channel blocker; ARB: Angiotensin Receptor Blocker.

OCTA Parameters	Medication Groups			*One-Way ANOVA* *p*-Value
	ACEI + Statins	CCB + Statins	ACEI/ARB + Statins	
	(N = 34)	(N = 30)	(N = 34)	
	M ± SD	M ± SD	M ± SD	
NFA Area *	0.35 ± 0.10	0.42 ± 0.12	0.51 ± 0.15	**<0.001**
FAZ Area *	0.37 ± 0.16	0.59 ± 0.22	0.62 ± 0.17	**<0.001**
FAZ Perimeter	3.41 ± 2.21	3.61 ± 2.39	3.60 ± 1.86	0.910
FAZ Circularity *	0.50 ± 0.14	0.42 ± 0.14	0.51 ± 0.16	**0.021**
VFA Area	3.15 ± 0.02	3.15 ± 0.01	3.12 ± 0.12	0.144
VFA Flow Area *	1.43 ± 0.30	1.38 ± 0.36	1.24 ± 0.29	**0.038**
Density Total *	36.10 ± 1.36	35.11 ± 2.03	33.15 ± 5.28	**0.002**
Density ETDRS *	35.14 ± 2.11	34.01 ± 2.29	31.92 ± 5.89	**0.004**
Skeleton Total	20.66 ± 1.45	21.17 ± 0.83	20.64 ± 0.96	0.118
Skeleton ETDRS	20.78 ± 1.48	21.16 ± 0.98	20.44 ± 1.57	0.115

* statistically relevant

**Table 3:** Bonferroni post hoc test. OCTA: optical coherence tomography angiography; FAZ: foveal avascular zone; NFA: non-flow area; VFA: vascular flow area; ETDRS: Early Treatment of Diabetic Retinopathy Study; ACEI: Angiotensin-converting enzyme inhibitor; CCB: Calcium channel blockers; ARB: Angiotensin Receptor Blocker.

OCTA Parameters	Multiple Comparisons		*p*-Value
NFA Area	ACEI/ARB + statins	ACEI + statins	**<0.001**
		CCB + statins	**0.010**
FAZ Area	ACEI + statins	CCB + statins	**<0.001**
		ACEI/ARB + statins	**<0.001**
FAZ Circularity	CCB + statins	ACEI + statins	0.066
		ACEI/ARB + statins	**0.033**
VFA Flow Area	ACEI + statins	CCB + statins	1.000
		ACEI/ARB + statins	**0.040**
Density Total	ACEI + statins	CCB + statins	0.746
		ACEI/ARB + statins	**0.002**
Density ETDRS	ACEI + statins	CCB + statins	0.742
		ACEI/ARB + statins	**0.003**

**Table 4:** Multivariate tests *^a^*. SBP: systolic blood pressure; DBP: diastolic blood pressure; HDL: high-density lipoprotein; LDL: low-density lipoprotein. a. Design: SBP + DBP + total cholesterol + HDL + LDL + triglycerides + disease duration + medication groups; b. Exact statistic.

Effect		Value	F	Hypothesis df	Error df	*p*	Partial Eta Squared
SBP	Pillai’s Trace	0.116	1.032 ^b^	10	79	0.425	0.116
	Wilks’ Lambda	0.884	1.032 ^b^	10	79	0.425	0.116
	Hotelling’s Trace	0.131	1.032 ^b^	10	79	0.425	0.116
	Roy’s Largest Root	0.131	1.032 ^b^	10	79	0.425	0.116
DBP	Pillai’s Trace	0.097	0.848 ^b^	10	79	0.585	0.097
	Wilks’ Lambda	0.903	0.848 ^b^	10	79	0.585	0.097
	Hotelling’s Trace	0.107	0.848 ^b^	10	79	0.585	0.097
	Roy’s Largest Root	0.107	0.848 ^b^	10	79	0.585	0.097
Total cholesterol	Pillai’s Trace	0.230	2.357 ^b^	10	79	**0.017**	0.230
	Wilks’ Lambda	0.770	2.357 ^b^	10	79	**0.017**	0.230
	Hotelling’s Trace	0.298	2.357 ^b^	10	79	**0.017**	0.230
	Roy’s Largest Root	0.298	2.357 ^b^	10	79	**0.017**	0.230
HDL	Pillai’s Trace	0.131	1.191 ^b^	10	79	0.310	0.131
	Wilks’ Lambda	0.869	1.191 ^b^	10	79	0.310	0.131
	Hotelling’s Trace	0.151	1.191 ^b^	10	79	0.310	0.131
	Roy’s Largest Root	0.151	1.191 ^b^	10	79	0.310	0.131
LDL	Pillai’s Trace	0.194	1.907 ^b^	10	79	0.056	0.194
	Wilks’ Lambda	0.806	1.907 ^b^	10	79	0.056	0.194
	Hotelling’s Trace	0.241	1.907 ^b^	10	79	0.056	0.194
	Roy’s Largest Root	0.241	1.907 ^b^	10	79	0.056	0.194
Triglycerides	Pillai’s Trace	0.172	1.639 ^b^	10	79	0.111	0.172
	Wilks’ Lambda	0.828	1.639 ^b^	10	79	0.111	0.172
	Hotelling’s Trace	0.208	1.639 ^b^	10	79	0.111	0.172
	Roy’s Largest Root	0.208	1.639 ^b^	10	79	0.111	0.172
Disease duration	Pillai’s Trace	0.154	1.434 ^b^	10	79	0.181	0.154
	Wilks’ Lambda	0.846	1.434 ^b^	10	79	0.181	0.154
	Hotelling’s Trace	0.181	1.434 ^b^	10	79	0.181	0.154
	Roy’s Largest Root	0.181	1.434 ^b^	10	79	0.181	0.154
Medication groups	Pillai’s Trace	0.564	3.139	20	160	**<0.001**	0.282
	Wilks’ Lambda	0.512	3.139 ^b^	20	158	**<0.001**	0.284
	Hotelling’s Trace	0.805	3.138	20	156	**<0.001**	0.287
	Roy’s Largest Root	0.520	4.160	10	80	**<0.001**	0.342

**Table 5:** Tests of Between-Subjects Effects. SBP: systolic blood pressure; DBP: diastolic blood pressure; HDL: high-density lipoprotein; LDL: low-density lipoprotein; FAZ: foveal avascular zone; NFA: non-flow area; VFA: vascular flow area; ETDRS: Early Treatment of Diabetic Retinopathy Study.

Source	OCTA Parameters	Type III Sum of Squares	df	Mean Square	F	*p*	Partial Eta Squared
SBP	VFA Area	0.020	1	0.020	4.161	0.044	0.045
DBP	NFA Area	0.065	1	0.065	4.240	0.042	0.046
Total cholesterol	FAZ Perimeter	21.248	1	21.248	4.575	0.035	0.049
	VFA Flow Area	0.411	1	0.411	4.153	0.045	0.045
	Skeleton ETDRS	15.487	1	15.487	8.604	0.004	0.089
HDL	Density ETDRS	73.946	1	73.946	5.081	0.027	0.055
	Skeleton ETDRS	11.863	1	11.863	6.591	0.012	0.070
LDL	Skeleton ETDRS	10.633	1	10.633	5.908	0.017	0.063
Triglycerides	Density Total	88.044	1	88.044	7.949	0.006	0.083
	Density ETDRS	114.863	1	114.863	7.892	0.006	0.082
Disease duration	Skeleton Total	6.335	1	6.335	5.331	0.023	0.057
Medication groups	NFA Area	0.139	2	0.070	4.577	0.013	0.094
	FAZ Area	0.754	2	0.377	11.031	<0.001	0.200
	FAZ Circularity	0.161	2	0.081	3.790	0.026	0.079
	VFA Flow Area	0.625	2	0.313	3.163	0.047	0.067
	Skeleton ETDRS	10.946	2	5.473	3.041	0.053	0.065

**Table 6:** Pairwise comparisons. * The mean difference is significant at the 0.05 level. b. Adjustment for multiple comparisons: Bonferroni.

Dependent Variable	Medication Groups		Mean Difference (I-J)	Std. Error	*p*	95% Confidence Interval for Difference ^b^	
						Lower Bound	Upper Bound
NFA Area	ACEI/ARB + statins	ACEI + statins	0.149 *	0.049	**0.010**	0.028	0.269
		CCB + statins	0.080	0.040	0.151	−0.018	0.179
	ACEI + statins	ACEI/ARB + statins	−0.149 *	0.049	**0.010**	−0.269	−0.028
		CCB + statins	−0.068	0.034	0.145	−0.152	0.015
	CCB + statins	ACEI/ARB + statins	−0.080	0.040	0.151	−0.179	0.018
		ACEI + statins	0.068	0.034	0.145	−0.015	0.152
FAZ Area	ACEI/ARB + statins	ACEI + statins	0.255 *	0.074	**0.003**	0.075	0.436
		CCB + statins	0.019	0.060	1.000	−0.128	0.167
	ACEI + statins	ACEI/ARB + statins	−0.255 *	0.074	**0.003**	−0.436	−0.075
		CCB + statins	−0.236 *	0.051	**<0.001**	−0.361	−0.111
	CCB + statins	ACEI/ARB + statins	−0.019	0.060	1.000	−0.167	0.128
		ACEI + statins	0.236 *	0.051	**<0.001**	0.111	0.361
FAZ Circularity	ACEI/ARB + statins	ACEI + statins	−0.016	0.058	1.000	−0.159	0.126
		CCB + statins	0.080	0.048	0.290	−0.036	0.196
	ACEI + statins	ACEI/ARB + statins	0.016	0.058	1.000	−0.126	0.159
		CCB + statins	0.096	0.040	**0.058**	−0.002	0.195
	CCB + statins	ACEI/ARB + statins	−0.080	0.048	0.290	−0.196	0.036
		ACEI + statins	−0.096	0.040	**0.058**	−0.195	0.002
VFA Flow Area	ACEI/ARB + statins	ACEI + statins	−0.306	0.126	**0.051**	−0.613	0.001
		CCB + statins	−0.226	0.103	0.091	−0.477	0.025
	ACEI + statins	ACEI/ARB + statins	0.306	0.126	**0.051**	−0.001	0.613
		CCB + statins	0.080	0.087	1.000	−0.133	0.293
	CCB + statins	ACEI/ARB + statins	0.226	0.103	0.091	−0.025	0.477
		ACEI + statins	−0.080	0.087	1.000	−0.293	0.133
Skeleton ETDRS	ACEI/ARB + statins	ACEI + statins	−0.979	0.536	0.214	−2.288	0.330
		CCB + statins	−10.081 *	0.439	**0.047**	−2.152	−0.011
	ACEI + statins	ACEI/ARB + statins	0.979	0.536	0.214	−0.330	2.288
		CCB + statins	−0.103	0.372	1.000	−1.010	0.805
	CCB + statins	ACEI/ARB + statins	10.081 *	0.439	**0.047**	0.011	2.152
		ACEI + statins	0.103	0.372	1.000	−0.805	1.010

**Table 7:** Pearson and Spearman correlations in the ACEI + statins treatment group. * *p* < 0.05, ** *p* < 0.01, *** *p* < 0.001. FAZ: foveal avascular zone; NFA: non-flow area; VFA: vascular flow area; ETDRS: Early Treatment of Diabetic Retinopathy Study; SBP: systolic blood pressure; DBP: diastolic blood pressure; HDL: high-density lipoprotein; LDL: low-density lipoprotein.

OCTA Parameters, Carotid Doppler Ultrasound Parameter and Clinical Values			Shapiro-Wilk	Pearson		Spearman	
			*p*	r	*p*	rho	*p*
NFA Area	-	Total cholesterol	0.027	0.477 **	0.004	0.541 ***	<0.001
FAZ Area	-	FAZ Perimeter	<0.001	**0.315**	0.069	0.417 *	0.014
FAZ Area	-	Carotid plaque	<0.001	0.540 ***	<0.001	0.526 **	0.001
FAZ Perimeter	-	FAZ Circularity	<0.001	−0.545 ***	<0.001	−0.530 **	0.001
FAZ Perimeter	-	DBP	<0.001	0.413 *	0.015	0.455 **	0.007
FAZ Circularity	-	SBP	0.009	−0.345 *	0.045	−0.220	0.211
VFA Area	-	Carotid plaque	<0.001	−0.409 *	0.016	−0.430 *	0.011
Density Total	-	Density ETDRS	0.127	0.377 *	0.028	0.511 **	0.002
Skeleton Total	-	Skeleton ETDRS	0.204	−0.449 **	0.008	−0.511 **	0.002
SBP	-	DBP	<0.001	0.484 **	0.004	0.668 ***	<0.001
SBP	-	HDL	0.010	−0.459 **	0.006	**−0.214**	0.224
DBP	-	HDL	0.008	−0.455 **	0.007	−0.347 *	0.044
Total cholesterol	-	HDL	0.028	0.277	0.112	0.370 *	0.031
Total cholesterol	-	LDL	<0.001	0.808 ***	<0.001	0.667 ***	<0.001

**Table 8:** The CCB + statins treatment group. Pearson and Spearman correlations. * *p* < 0.05, ** *p* < 0.01, *** *p* < 0.001. FAZ: foveal avascular zone; NFA: non-flow area; VFA: vascular flow area; ETDRS: Early Treatment of Diabetic Retinopathy Study; SBP: systolic blood pressure; DBP: diastolic blood pressure; HDL: high-density lipoprotein; LDL: low-density lipoprotein.

OCTA Parameters, Carotid Doppler Ultrasound Parameter and Clinical Values			Shapiro–Wilk	Pearson		Spearman	
			*p*	r	*p*	rho	*p*
NFA Area	-	VFA Area	0.778	0.319	0.086	0.419 *	0.021
NFA Area	-	DBP	0.003	0.439 *	0.015	0.406 *	0.026
FAZ Perimeter	-	Skeleton Total	<0.001	−0.338	0.068	−0.450 *	0.013
FAZ Perimeter	-	Skeleton ETDRS	<0.001	−0.295	0.114	−0.437 *	0.016
FAZ Perimeter	-	SBP	<0.001	−0.425 *	0.019	−0.270	0.148
FAZ Perimeter	-	DBP	<0.001	−0.367 *	0.046	−0.300	0.107
FAZ Circularity	-	Carotid plaque	<0.001	−0.406 *	0.026	−0.409 *	0.025
Density Total	-	Carotid plaque	0.009	0.400 *	0.028	0.389 *	0.034
Skeleton Total	-	Skeleton ETDRS	0.002	0.952 ***	<0.001	0.931 ***	<0.001
Skeleton Total	-	SBP	0.003	0.413 *	0.023	0.381 *	0.038
Skeleton Total	-	DBP	<0.001	0.393 *	0.032	0.361 *	0.050
Skeleton ETDRS	-	SBP	<0.001	0.392 *	0.032	0.341	0.065
Skeleton ETDRS	-	DBP	<0.001	0.388 *	0.034	0.400 *	0.028
SBP	-	DBP	<0.001	0.503 **	0.005	0.394 *	0.031
SBP	-	Total cholesterol	0.026	0.379 *	0.039	0.325	0.080
Total cholesterol	-	HDL	<0.001	0.452 *	0.012	0.514 **	0.004
Total cholesterol	-	LDL	<0.001	0.836 ***	<0.001	0.868 ***	<0.001
HDL	-	LDL	<0.001	0.278	0.136	0.375 *	0.041

**Table 9:** Pearson and Spearman correlations in the ACEI/ARB + statins treatment group. * *p* < 0.05, ** *p* < 0.01, *** *p* < 0.001. FAZ: foveal avascular zone; NFA: non-flow area; VFA: vascular flow area; ETDRS: Early Treatment of Diabetic Retinopathy Study; SBP: systolic blood pressure; DBP: diastolic blood pressure; HDL: high-density lipoprotein; LDL: low-density lipoprotein.

OCTA Parameters, Carotid Doppler Ultrasound Parameter and Clinical Values			Shapiro–Wilk	Pearson		Spearman	
			*p*	r	*p*	rho	*p*
NFA Area	-	VFA Area	<0.001	**−0.248**	0.158	−0.420 *	0.013
NFA Area	-	Carotid plaque	<0.001	−0.558 ***	<0.001	−0.555 ***	<0.001
FAZ Area	-	Density Total	<0.001	−0.412 *	0.015	−0.447 **	0.008
FAZ Area	-	Skeleton ETDRS	0.273	−0.355 *	0.040	−0.281	0.108
FAZ Area	-	Carotid plaque	<0.001	0.405 *	0.017	0.394 *	0.021
FAZ Perimeter	-	FAZ Circularity	<0.001	−0.505 **	0.002	−0.593 ***	<0.001
FAZ Perimeter	-	Density Total	<0.001	−0.290	0.096	−0.536 **	0.001
FAZ Perimeter	-	Density ETDRS	<0.001	−0.421 *	0.013	−0.546 ***	<0.001
FAZ Circularity	-	Total cholesterol	<0.001	0.516 **	0.002	0.449 **	0.008
FAZ Circularity	-	LDL	0.002	0.444 **	0.009	0.343 *	0.047
VFA Area	-	SBP	<0.001	−0.330	0.057	−0.514 **	0.002
VFA Area	-	HDL	<0.001	0.242	0.168	0.395 *	0.021
Density Total	-	Density ETDRS	<0.001	0.735 ***	<0.001	0.710 ***	<0.001
Density Total	-	Skeleton ETDRS	<0.001	0.293	0.093	0.439 **	0.009
Density Total	-	Carotid plaque	<0.001	−0.527 **	0.001	−0.509 **	0.002
Density ETDRS	-	Skeleton Total	<0.001	−0.451 **	0.007	−0.420 *	0.013
Density ETDRS	-	Skeleton ETDRS	<0.001	0.393 *	0.022	0.561 ***	<0.001
Skeleton Total	-	Total cholesterol	<0.001	0.371 *	0.031	0.348 *	0.044
Skeleton Total	-	LDL	0.001	0.337	0.052	0.396 *	0.020
SBP	-	DBP	<0.001	0.434 *	0.010	0.420 *	0.013
SBP	-	HDL	<0.001	−0.461 **	0.006	−0.479 **	0.004
DBP	-	Total cholesterol	<0.001	0.374 *	0.029	0.290	0.096
DBP	-	LDL	<0.001	0.362 *	0.035	0.304	0.080
Total cholesterol	-	LDL	0.001	0.974 ***	<0.001	0.939 ***	<0.001

## Data Availability

The data that support the findings of the present study are available from the corresponding author upon reasonable request.

## Supplementary Materials

The following supporting information can be downloaded at: https://www.mdpi.com/article/10.3390/life16030436/s1, Table S1. Correlations in the ACEI treatment group; Table S2. Correlations in the CCB treatment group; Table S3. Correlations in the ACEI/ARB treatment group; Table S4. Correlations among all treatment groups; Table S5. Demographic and ultrasound parameters; Table S6. Shapiro–Wilk test; Table S7. Brown–Forsyth Robust Tests of Equality of Means; Table S8. Kruskal–Wallis test; Table S9. Dunn’s post hoc comparisons; Table S10. All therapy lots. Pearson and Spearman correlations; Table S11. Estimated Marginal Means and pairwise comparisons.
