# Supplementary material for: Retinal Dysfunction in Hypertensive Patients with Atherosclerotic Plaque Detected by Carotid Doppler Ultrasound: An Optical Coherence Tomography Angiography Assessment
Source: Life (Basel). 2026 Mar 9;16(3):436. doi: 10.3390/life16030436 (PMC13028594; doi:10.3390/life16030436)
Supplement: Supplementary file 1 [file life-16-00436-s001.zip › life-4137187-supplementary.pdf]

Supplementary Materials

Spearman and Pearson correlations calculated among OCTA parameters, carotid Doppler ultrasound and clinical measurements in all therapy groups are displayed in Tables S1, S2, S3 and S4.

Table S1. Correlations in the ACEI treatment group. * p < 0.05, ** p < .001, *** p < 0.001.

**OCTA parameters, carotid Doppler ultrasound parameter and clinical values**			**Shapiro-Wilk**	**Pearson**		**Spearman**	
			**p**	**r**	**p**	**rho**	**p**
NFA Area	-	FAZ Area	0.026	0.029	0.870	0.078	0.661
NFA Area	-	FAZ Perimeter	< 0.001	-0.034	0.848	-0.041	0.817
NFA Area	-	FAZ Circularity	0.106	-0.052	0.770	-0.110	0.534
NFA Area	-	VFA Area	0.009	-0.082	0.645	-0.081	0.647
NFA Area	-	VFA Flow Area	0.558	-0.287	0.099	-0.223	0.205
NFA Area	-	Density Total	0.039	0.001	0.997	-0.011	0.949
NFA Area	-	Density ETDRS	0.226	-0.237	0.178	-0.178	0.313
NFA Area	-	Skeleton Total	0.023	0.054	0.760	0.082	0.645
NFA Area	-	Skeleton ETDRS	0.164	-0.052	0.771	0.002	0.993
NFA Area	-	SBP	0.003	0.034	0.849	0.098	0.580
NFA Area	-	DBP	< 0.001	0.098	0.580	0.145	0.412
NFA Area	-	Total cholesterol	0.027	**0.477****	0.004	**0.541*****	< 0.001
NFA Area	-	HDL	0.084	0.151	0.394	0.187	0.289
NFA Area	-	LDL	0.064	0.288	0.099	0.302	0.083
NFA Area	-	Carotid plaque	< 0.001	-0.096	0.591	-0.106	0.552
FAZ Area	-	FAZ Perimeter	< 0.001	0.315	0.069	**0.417***	0.014
FAZ Area	-	FAZ Circularity	0.165	0.117	0.511	0.119	0.501
FAZ Area	-	VFA Area	0.012	-0.120	0.500	-0.124	0.485
FAZ Area	-	VFA Flow Area	0.443	-0.334	0.054	-0.335	0.053
FAZ Area	-	Density Total	0.114	-0.133	0.453	-0.082	0.643
FAZ Area	-	Density ETDRS	0.189	-0.178	0.315	-0.155	0.381
FAZ Area	-	Skeleton Total	0.033	0.059	0.739	0.097	0.584
FAZ Area	-	Skeleton ETDRS	0.281	-0.133	0.453	-0.165	0.352
FAZ Area	-	SBP	0.005	0.121	0.497	0.072	0.686
FAZ Area	-	DBP	0.002	0.303	0.081	0.252	0.150
FAZ Area	-	Total cholesterol	0.015	0.057	0.749	0.065	0.715
FAZ Area	-	HDL	0.119	-0.081	0.651	0.063	0.722
FAZ Area	-	LDL	0.025	0.009	0.959	-0.088	0.621
FAZ Area	-	Carotid plaque	< 0.001	**0.540*****	< 0.001	**0.526****	0.001
FAZ Perimeter	-	FAZ Circularity	< 0.001	**-0.545*****	< 0.001	**-0.530****	0.001
FAZ Perimeter	-	VFA Area	< 0.001	0.090	0.614	0.188	0.287
FAZ Perimeter	-	VFA Flow Area	< 0.001	-0.139	0.433	-0.122	0.494
FAZ Perimeter	-	Density Total	< 0.001	0.044	0.804	0.158	0.372
FAZ Perimeter	-	Density ETDRS	< 0.001	-0.209	0.235	0.009	0.961
FAZ Perimeter	-	Skeleton Total	< 0.001	0.037	0.833	0.085	0.632
FAZ Perimeter	-	Skeleton ETDRS	< 0.001	-0.042	0.813	-0.051	0.776

FAZ Perimeter	-	SBP	< 0.001	0.315	0.070	0.334	0.053
FAZ Perimeter	-	DBP	< 0.001	**0.413***	0.015	**0.455****	0.007
FAZ Perimeter	-	Total cholesterol	< 0.001	0.289	0.097	0.263	0.133
FAZ Perimeter	-	HDL	< 0.001	-0.138	0.435	0.031	0.861
FAZ Perimeter	-	LDL	< 0.001	0.020	0.910	-0.016	0.927
FAZ Perimeter	-	Carotid plaque	< 0.001	-0.062	0.726	0.075	0.671
FAZ Circularity	-	VFA Area	0.165	0.133	0.452	-0.016	0.931
FAZ Circularity	-	VFA Flow Area	0.494	-0.037	0.837	-0.074	0.677
FAZ Circularity	-	Density Total	0.065	-0.274	0.117	-0.199	0.258
FAZ Circularity	-	Density ETDRS	0.412	0.017	0.925	0.020	0.910
FAZ Circularity	-	Skeleton Total	0.253	-0.128	0.470	-0.209	0.235
FAZ Circularity	-	Skeleton ETDRS	0.532	0.170	0.337	0.175	0.322
FAZ Circularity	-	SBP	0.009	**-0.345***	0.045	-0.220	0.211
FAZ Circularity	-	DBP	0.007	-0.294	0.092	-0.259	0.139
FAZ Circularity	-	Total cholesterol	0.257	-0.255	0.146	-0.209	0.235
FAZ Circularity	-	HDL	0.235	0.161	0.364	0.106	0.551
FAZ Circularity	-	LDL	0.170	-0.077	0.666	-0.052	0.770
FAZ Circularity	-	Carotid plaque	< 0.001	-0.075	0.674	-0.067	0.708
VFA Area	-	VFA Flow Area	0.031	0.013	0.941	0.070	0.695
VFA Area	-	Density Total	0.014	-0.037	0.835	-0.125	0.482
VFA Area	-	Density ETDRS	0.021	0.023	0.899	0.021	0.907
VFA Area	-	Skeleton Total	0.007	0.021	0.908	-0.013	0.943
VFA Area	-	Skeleton ETDRS	0.047	-0.172	0.331	-0.163	0.356
VFA Area	-	SBP	0.006	-0.194	0.271	0.055	0.759
VFA Area	-	DBP	< 0.001	-0.002	0.990	0.059	0.739
VFA Area	-	Total cholesterol	0.004	-0.011	0.949	0.100	0.574
VFA Area	-	HDL	0.033	0.071	0.689	0.026	0.883
VFA Area	-	LDL	0.005	-0.288	0.099	-0.284	0.103
VFA Area	-	Carotid plaque	< 0.001	**-0.409***	0.016	**-0.430***	0.011
VFA Flow Area	-	Density Total	0.353	0.180	0.310	0.154	0.385
VFA Flow Area	-	Density ETDRS	0.446	0.198	0.262	0.212	0.229
VFA Flow Area	-	Skeleton Total	0.144	-0.233	0.184	-0.234	0.182
VFA Flow Area	-	Skeleton ETDRS	0.605	0.198	0.261	0.174	0.324
VFA Flow Area	-	SBP	0.014	-0.069	0.700	-0.144	0.416
VFA Flow Area	-	DBP	0.003	-0.069	0.697	-0.147	0.407
VFA Flow Area	-	Total cholesterol	0.224	-0.226	0.198	-0.249	0.155
VFA Flow Area	-	HDL	0.220	0.109	0.540	0.032	0.858
VFA Flow Area	-	LDL	0.156	-0.058	0.745	-0.049	0.782
VFA Flow Area	-	Carotid plaque	< 0.001	-0.286	0.102	-0.302	0.083
Density Total	-	Density ETDRS	0.127	**0.377***	0.028	**0.511****	0.002
Density Total	-	Skeleton Total	0.058	0.024	0.894	0.086	0.630
Density Total	-	Skeleton ETDRS	0.353	0.231	0.189	0.233	0.185
Density Total	-	SBP	0.008	-0.079	0.655	-0.092	0.604

Density Total	-	DBP	0.001	-0.173	0.328	-0.173	0.327
Density Total	-	Total cholesterol	0.033	-0.060	0.737	-0.092	0.606
Density Total	-	HDL	0.227	0.149	0.401	0.071	0.691
Density Total	-	LDL	0.053	-0.051	0.774	-0.036	0.838
Density Total	-	Carotid plaque	< 0.001	0.101	0.570	0.142	0.423
Density ETDRS	-	Skeleton Total	0.119	-0.107	0.547	-0.095	0.593
Density ETDRS	-	Skeleton ETDRS	0.666	0.256	0.143	0.255	0.146
Density ETDRS	-	SBP	0.014	-0.053	0.765	-0.081	0.651
Density ETDRS	-	DBP	0.003	-0.162	0.360	-0.214	0.223
Density ETDRS	-	Total cholesterol	0.062	-0.242	0.167	-0.301	0.083
Density ETDRS	-	HDL	0.250	0.094	0.597	-0.017	0.923
Density ETDRS	-	LDL	0.083	-0.047	0.793	-0.085	0.633
Density ETDRS	-	Carotid plaque	< 0.001	0.061	0.733	0.066	0.709
Skeleton Total	-	Skeleton ETDRS	0.204	**-0.449****	0.008	**-0.511****	0.002
Skeleton Total	-	SBP	0.004	0.016	0.930	0.057	0.748
Skeleton Total	-	DBP	< 0.001	0.095	0.592	0.108	0.543
Skeleton Total	-	Total cholesterol	0.028	-0.078	0.661	-0.095	0.593
Skeleton Total	-	HDL	0.075	-0.085	0.635	-0.063	0.724
Skeleton Total	-	LDL	0.053	-0.264	0.132	-0.270	0.123
Skeleton Total	-	Carotid plaque	< 0.001	0.217	0.217	0.197	0.265
Skeleton ETDRS	-	SBP	0.030	-0.311	0.074	-0.216	0.221
Skeleton ETDRS	-	DBP	0.006	-0.176	0.318	-0.135	0.446
Skeleton ETDRS	-	Total cholesterol	0.119	-0.008	0.964	-0.033	0.853
Skeleton ETDRS	-	HDL	0.472	0.137	0.440	-0.083	0.642
Skeleton ETDRS	-	LDL	0.328	0.301	0.084	0.243	0.166
Skeleton ETDRS	-	Carotid plaque	< 0.001	-0.091	0.610	-0.106	0.552
SBP	-	DBP	< 0.001	**0.484****	0.004	**0.668*****	< 0.001
SBP	-	Total cholesterol	< 0.001	0.264	0.132	0.262	0.134
SBP	-	HDL	0.010	**-0.459****	0.006	-0.214	0.224
SBP	-	LDL	0.005	0.097	0.587	-0.049	0.782
SBP	-	Carotid plaque	< 0.001	0.101	0.568	0.012	0.946
DBP	-	Total cholesterol	< 0.001	0.271	0.122	0.291	0.095
DBP	-	HDL	0.008	**-0.455****	0.007	**-0.347***	0.044
DBP	-	LDL	< 0.001	0.103	0.561	0.099	0.576
DBP	-	Carotid plaque	< 0.001	0.131	0.460	0.079	0.658
Total cholesterol	-	HDL	0.028	0.277	0.112	**0.370***	0.031
Total cholesterol	-	LDL	< 0.001	**0.808*****	< 0.001	**0.667*****	< 0.001
Total cholesterol	-	Carotid plaque	< 0.001	-0.118	0.506	-0.218	0.216
HDL	-	LDL	0.184	0.338	0.050	0.260	0.138
HDL	-	Carotid plaque	< 0.001	-0.168	0.342	-0.169	0.339
LDL	-	Carotid plaque	< 0.001	0.084	0.635	0.036	0.839

Table S2. Correlations in the CCB treatment group. * p < 0.05, ** p < .001, *** p < 0.001.

OCTA parameters, carotid Doppler ultrasound parameter and clinical values			Shapiro-Wilk	Pearson		Spearman	
			p	r	p	rho	p
NFA Area	-	FAZ Area	0.220	0.015	0.939	0.060	0.752
NFA Area	-	FAZ Perimeter	< 0.001	0.189	0.318	0.053	0.782
NFA Area	-	FAZ Circularity	0.945	0.000	0.998	0.044	0.816
NFA Area	-	VFA Area	0.778	0.319	0.086	**0.419***	0.021
NFA Area	-	VFA Flow Area	0.978	0.047	0.806	0.025	0.897
NFA Area	-	Density Total	0.089	-0.020	0.915	-0.052	0.784
NFA Area	-	Density ETDRS	0.216	-0.070	0.715	-0.035	0.855
NFA Area	-	Skeleton Total	0.524	0.047	0.804	-0.034	0.857
NFA Area	-	Skeleton ETDRS	0.113	0.097	0.609	0.054	0.777
NFA Area	-	SBP	0.011	0.031	0.870	0.087	0.646
NFA Area	-	DBP	0.003	**0.439***	0.015	**0.406***	0.026
NFA Area	-	Total cholesterol	0.276	-0.031	0.872	0.064	0.736
NFA Area	-	HDL	0.138	0.327	0.077	0.350	0.058
NFA Area	-	LDL	0.036	0.000	0.998	0.097	0.609
NFA Area	-	Carotid plaque	< 0.001	-0.007	0.972	-0.031	0.870
FAZ Area	-	FAZ Perimeter	< 0.001	0.356	0.054	0.182	0.336
FAZ Area	-	FAZ Circularity	0.288	0.116	0.542	0.095	0.617
FAZ Area	-	VFA Area	0.046	-0.093	0.626	0.077	0.686
FAZ Area	-	VFA Flow Area	0.291	-0.140	0.462	-0.147	0.439
FAZ Area	-	Density Total	0.010	-0.003	0.989	0.028	0.881
FAZ Area	-	Density ETDRS	0.026	-0.012	0.951	0.008	0.966
FAZ Area	-	Skeleton Total	0.130	-0.164	0.386	-0.153	0.418
FAZ Area	-	Skeleton ETDRS	0.017	-0.162	0.393	-0.217	0.249
FAZ Area	-	SBP	0.002	0.050	0.793	0.051	0.787
FAZ Area	-	DBP	< 0.001	-0.062	0.744	0.037	0.846
FAZ Area	-	Total cholesterol	0.070	0.131	0.491	0.178	0.345
FAZ Area	-	HDL	0.013	0.008	0.968	0.083	0.661
FAZ Area	-	LDL	0.004	0.107	0.573	0.143	0.452
FAZ Area	-	Carotid plaque	< 0.001	-0.097	0.612	-0.148	0.436
FAZ Perimeter	-	FAZ Circularity	< 0.001	-0.235	0.212	-0.286	0.125
FAZ Perimeter	-	VFA Area	< 0.001	-0.189	0.318	-0.185	0.327
FAZ Perimeter	-	VFA Flow Area	< 0.001	-0.162	0.391	-0.063	0.740
FAZ Perimeter	-	Density Total	< 0.001	-0.059	0.756	-0.237	0.208
FAZ Perimeter	-	Density ETDRS	< 0.001	-0.307	0.099	-0.193	0.306
FAZ Perimeter	-	Skeleton Total	< 0.001	-0.338	0.068	**-0.450***	0.013
FAZ Perimeter	-	Skeleton ETDRS	< 0.001	-0.295	0.114	**-0.437***	0.016
FAZ Perimeter	-	SBP	< 0.001	**-0.425***	0.019	-0.270	0.148
FAZ Perimeter	-	DBP	< 0.001	**-0.367***	0.046	-0.300	0.107
FAZ Perimeter	-	Total cholesterol	< 0.001	0.157	0.407	0.345	0.062

FAZ Perimeter	-	HDL	< 0.001	0.153	0.421	0.137	0.469
FAZ Perimeter	-	LDL	< 0.001	0.060	0.755	0.193	0.308
FAZ Perimeter	-	Carotid plaque	< 0.001	0.125	0.510	0.206	0.275
FAZ Circularity	-	VFA Area	0.916	0.243	0.196	0.200	0.290
FAZ Circularity	-	VFA Flow Area	0.988	0.103	0.588	0.099	0.603
FAZ Circularity	-	Density Total	0.070	0.229	0.223	0.194	0.305
FAZ Circularity	-	Density ETDRS	0.332	0.138	0.468	0.178	0.347
FAZ Circularity	-	Skeleton Total	0.623	0.012	0.949	0.065	0.732
FAZ Circularity	-	Skeleton ETDRS	0.120	-0.093	0.626	-0.017	0.929
FAZ Circularity	-	SBP	0.010	0.304	0.103	0.309	0.097
FAZ Circularity	-	DBP	0.002	0.185	0.327	0.060	0.754
FAZ Circularity	-	Total cholesterol	0.364	0.067	0.724	0.127	0.502
FAZ Circularity	-	HDL	0.112	0.136	0.475	0.159	0.402
FAZ Circularity	-	LDL	0.054	0.069	0.717	0.116	0.541
FAZ Circularity	-	Carotid plaque	< 0.001	**-0.406***	0.026	**-0.409***	0.025
VFA Area	-	VFA Flow Area	0.953	-0.222	0.238	-0.136	0.475
VFA Area	-	Density Total	0.009	0.033	0.863	0.143	0.452
VFA Area	-	Density ETDRS	0.077	-0.128	0.502	0.067	0.725
VFA Area	-	Skeleton Total	0.671	0.354	0.055	0.310	0.096
VFA Area	-	Skeleton ETDRS	0.122	0.318	0.087	0.329	0.076
VFA Area	-	SBP	0.001	0.049	0.798	0.076	0.690
VFA Area	-	DBP	0.002	0.222	0.239	0.249	0.184
VFA Area	-	Total cholesterol	0.019	0.002	0.993	0.017	0.930
VFA Area	-	HDL	0.026	0.216	0.251	0.269	0.151
VFA Area	-	LDL	0.005	-0.051	0.788	0.057	0.765
VFA Area	-	Carotid plaque	< 0.001	0.008	0.967	-0.008	0.966
VFA Flow Area	-	Density Total	0.091	-0.319	0.086	-0.348	0.060
VFA Flow Area	-	Density ETDRS	0.396	0.135	0.477	0.077	0.685
VFA Flow Area	-	Skeleton Total	0.696	0.088	0.642	0.177	0.349
VFA Flow Area	-	Skeleton ETDRS	0.161	0.041	0.829	0.159	0.400
VFA Flow Area	-	SBP	0.021	0.265	0.157	0.217	0.248
VFA Flow Area	-	DBP	0.003	0.202	0.284	0.270	0.149
VFA Flow Area	-	Total cholesterol	0.467	0.047	0.804	0.018	0.925
VFA Flow Area	-	HDL	0.187	-0.017	0.928	0.035	0.853
VFA Flow Area	-	LDL	0.068	0.153	0.420	0.213	0.259
VFA Flow Area	-	Carotid plaque	< 0.001	0.012	0.950	-0.066	0.728
Density Total	-	Density ETDRS	0.010	0.181	0.340	0.186	0.325
Density Total	-	Skeleton Total	0.022	-0.112	0.557	-0.128	0.502
Density Total	-	Skeleton ETDRS	0.003	-0.089	0.639	-0.132	0.487
Density Total	-	SBP	0.002	-0.277	0.138	-0.271	0.148
Density Total	-	DBP	< 0.001	-0.284	0.129	-0.283	0.130
Density Total	-	Total cholesterol	0.015	-0.093	0.626	-0.131	0.490
Density Total	-	HDL	0.032	0.232	0.217	0.199	0.291

Density Total	-	LDL	0.003	-0.220	0.243	-0.207	0.273
Density Total	-	Carotid plaque	0.009	**0.400***	0.028	**0.389***	0.034
Density ETDRS	-	Skeleton Total	0.058	0.093	0.626	0.170	0.368
Density ETDRS	-	Skeleton ETDRS	0.008	0.115	0.546	0.189	0.317
Density ETDRS	-	SBP	0.002	0.141	0.458	0.148	0.436
Density ETDRS	-	DBP	< 0.001	0.281	0.133	0.294	0.115
Density ETDRS	-	Total cholesterol	0.055	0.101	0.596	0.128	0.502
Density ETDRS	-	HDL	0.015	0.056	0.767	0.052	0.786
Density ETDRS	-	LDL	0.005	0.072	0.704	0.062	0.747
Density ETDRS	-	Carotid plaque	< 0.001	0.000	0.998	-0.086	0.653
Skeleton Total	-	Skeleton ETDRS	0.002	**0.952*****	< 0.001	**0.931*****	< 0.001
Skeleton Total	-	SBP	0.003	**0.413***	0.023	**0.381***	0.038
Skeleton Total	-	DBP	< 0.001	**0.393***	0.032	**0.361***	0.050
Skeleton Total	-	Total cholesterol	0.118	-0.044	0.817	-0.033	0.864
Skeleton Total	-	HDL	0.040	-0.119	0.530	-0.070	0.714
Skeleton Total	-	LDL	0.008	0.068	0.719	0.176	0.353
Skeleton Total	-	Carotid plaque	< 0.001	0.129	0.496	0.082	0.667
Skeleton ETDRS	-	SBP	< 0.001	**0.392***	0.032	0.341	0.065
Skeleton ETDRS	-	DBP	< 0.001	**0.388***	0.034	**0.400***	0.028
Skeleton ETDRS	-	Total cholesterol	0.013	-0.083	0.663	-0.146	0.443
Skeleton ETDRS	-	HDL	0.008	-0.105	0.581	-0.024	0.901
Skeleton ETDRS	-	LDL	0.002	0.063	0.743	0.063	0.739
Skeleton ETDRS	-	Carotid plaque	< 0.001	0.155	0.414	0.078	0.682
SBP	-	DBP	< 0.001	**0.503****	0.005	**0.394***	0.031
SBP	-	Total cholesterol	0.026	0.379*	0.039	0.325	0.080
SBP	-	HDL	< 0.001	0.036	0.852	-0.061	0.747
SBP	-	LDL	0.002	0.301	0.106	0.296	0.112
SBP	-	Carotid plaque	< 0.001	0.048	0.799	0.126	0.507
DBP	-	Total cholesterol	< 0.001	0.068	0.719	0.153	0.420
DBP	-	HDL	< 0.001	0.257	0.170	0.325	0.079
DBP	-	LDL	< 0.001	0.203	0.282	0.205	0.278
DBP	-	Carotid plaque	< 0.001	0.004	0.983	0.000	1000.000
Total cholesterol	-	HDL	< 0.001	**0.452***	0.012	**0.514****	0.004
Total cholesterol	-	LDL	< 0.001	**0.836*****	< 0.001	**0.868*****	< 0.001
Total cholesterol	-	Carotid plaque	< 0.001	0.263	0.160	0.234	0.214
HDL	-	LDL	< 0.001	0.278	0.136	**0.375***	0.041
HDL	-	Carotid plaque	< 0.001	-0.159	0.401	-0.187	0.323
LDL	-	Carotid plaque	< 0.001	0.172	0.364	0.171	0.366

Table S3. Correlations in the ACEI/ARB treatment group. * p < 0.05, ** p < .001, *** p < 0.001.

OCTA parameters, carotid Doppler ultrasound parameter and clinical values			Shapiro-Wilk	Pearson		Spearman	
			p	**r**	p	**rho**	p
NFA Area	-	FAZ Area	0.000	-0.253	0.150	-0.260	0.137
NFA Area	-	FAZ Perimeter	< 0.001	0.104	0.557	0.140	0.428
NFA Area	-	FAZ Circularity	0.067	0.012	0.945	0.034	0.849
NFA Area	-	VFA Area	< 0.001	-0.248	0.158	**-0.420***	0.013
NFA Area	-	VFA Flow Area	0.207	0.199	0.260	0.116	0.514
NFA Area	-	Density Total	< 0.001	0.176	0.319	0.289	0.097
NFA Area	-	Density ETDRS	< 0.001	-0.096	0.588	0.071	0.690
NFA Area	-	Skeleton Total	0.020	-0.128	0.472	-0.023	0.896
NFA Area	-	Skeleton ETDRS	0.128	0.029	0.870	0.152	0.391
NFA Area	-	SBP	0.003	0.248	0.158	0.210	0.232
NFA Area	-	DBP	< 0.001	0.266	0.129	0.089	0.617
NFA Area	-	Total cholesterol	< 0.001	-0.109	0.538	-0.183	0.301
NFA Area	-	HDL	0.002	-0.151	0.395	-0.255	0.145
NFA Area	-	LDL	0.003	-0.170	0.337	-0.264	0.131
NFA Area	-	Carotid plaque	< 0.001	**-0.558*****	< 0.001	**-0.555*****	< 0.001
FAZ Area	-	FAZ Perimeter	< 0.001	0.186	0.293	0.168	0.343
FAZ Area	-	FAZ Circularity	0.023	-0.005	0.978	0.033	0.853
FAZ Area	-	VFA Area	< 0.001	0.287	0.100	0.131	0.459
FAZ Area	-	VFA Flow Area	0.055	0.136	0.443	0.123	0.487
FAZ Area	-	Density Total	< 0.001	**-0.412***	0.015	**-0.447****	0.008
FAZ Area	-	Density ETDRS	< 0.001	-0.313	0.071	-0.294	0.091
FAZ Area	-	Skeleton Total	0.004	0.096	0.587	0.079	0.658
FAZ Area	-	Skeleton ETDRS	0.273	**-0.355***	0.040	-0.281	0.108
FAZ Area	-	SBP	0.002	-0.275	0.115	-0.234	0.183
FAZ Area	-	DBP	< 0.001	-0.074	0.678	-0.187	0.289
FAZ Area	-	Total cholesterol	< 0.001	0.095	0.592	0.084	0.636
FAZ Area	-	HDL	< 0.001	-0.022	0.902	0.003	0.987
FAZ Area	-	LDL	< 0.001	0.137	0.438	0.147	0.408
FAZ Area	-	Carotid plaque	< 0.001	**0.405***	0.017	**0.394***	0.021
FAZ Perimeter	-	FAZ Circularity	< 0.001	**-0.505****	0.002	**-0.593*****	< 0.001
FAZ Perimeter	-	VFA Area	< 0.001	-0.006	0.973	-0.141	0.427
FAZ Perimeter	-	VFA Flow Area	< 0.001	0.084	0.636	0.249	0.156
FAZ Perimeter	-	Density Total	< 0.001	-0.290	0.096	**-0.536****	0.001
FAZ Perimeter	-	Density ETDRS	< 0.001	**-0.421***	0.013	**-0.546*****	< 0.001
FAZ Perimeter	-	Skeleton Total	< 0.001	0.196	0.266	0.254	0.148
FAZ Perimeter	-	Skeleton ETDRS	0.002	-0.321	0.064	-0.220	0.211
FAZ Perimeter	-	SBP	< 0.001	0.059	0.741	0.060	0.734
FAZ Perimeter	-	DBP	< 0.001	0.153	0.387	-0.060	0.736
FAZ Perimeter	-	Total cholesterol	< 0.001	-0.123	0.487	-0.208	0.239

FAZ Perimeter	-	HDL	< 0.001	-0.192	0.276	-0.182	0.304
FAZ Perimeter	-	LDL	< 0.001	-0.056	0.755	-0.075	0.671
FAZ Perimeter	-	Carotid plaque	< 0.001	0.001	1000	0.140	0.429
FAZ Circularity	-	VFA Area	< 0.001	0.321	0.064	0.155	0.382
FAZ Circularity	-	VFA Flow Area	0.309	-0.035	0.844	-0.090	0.611
FAZ Circularity	-	Density Total	< 0.001	0.025	0.887	0.156	0.378
FAZ Circularity	-	Density ETDRS	< 0.001	0.085	0.633	0.286	0.101
FAZ Circularity	-	Skeleton Total	0.021	0.081	0.648	0.111	0.534
FAZ Circularity	-	Skeleton ETDRS	0.639	0.339	0.050	0.310	0.075
FAZ Circularity	-	SBP	0.002	0.010	0.957	0.081	0.651
FAZ Circularity	-	DBP	< 0.001	0.218	0.215	0.225	0.200
FAZ Circularity	-	Total cholesterol	< 0.001	**0.516****	0.002	**0.449****	0.008
FAZ Circularity	-	HDL	0.008	0.251	0.153	0.189	0.284
FAZ Circularity	-	LDL	0.002	**0.444****	0.009	**0.343***	0.047
FAZ Circularity	-	Carotid plaque	< 0.001	0.013	0.942	-0.012	0.945
VFA Area	-	VFA Flow Area	< 0.001	-0.054	0.763	0.049	0.785
VFA Area	-	Density Total	< 0.001	-0.197	0.264	-0.157	0.376
VFA Area	-	Density ETDRS	< 0.001	0.187	0.290	0.146	0.409
VFA Area	-	Skeleton Total	< 0.001	-0.086	0.627	0.022	0.902
VFA Area	-	Skeleton ETDRS	< 0.001	0.215	0.223	0.028	0.874
VFA Area	-	SBP	< 0.001	-0.330	0.057	**-0.514****	0.002
VFA Area	-	DBP	< 0.001	-0.120	0.500	-0.095	0.593
VFA Area	-	Total cholesterol	< 0.001	0.068	0.704	0.165	0.352
VFA Area	-	HDL	< 0.001	0.242	0.168	**0.395***	0.021
VFA Area	-	LDL	< 0.001	0.039	0.828	0.105	0.553
VFA Area	-	Carotid plaque	< 0.001	0.214	0.225	0.289	0.097
VFA Flow Area	-	Density Total	< 0.001	-0.206	0.242	-0.158	0.373
VFA Flow Area	-	Density ETDRS	< 0.001	-0.240	0.171	-0.109	0.538
VFA Flow Area	-	Skeleton Total	0.054	0.009	0.960	0.020	0.910
VFA Flow Area	-	Skeleton ETDRS	0.524	-0.071	0.691	-0.023	0.897
VFA Flow Area	-	SBP	0.005	0.134	0.451	0.080	0.654
VFA Flow Area	-	DBP	< 0.001	0.261	0.136	0.148	0.402
VFA Flow Area	-	Total cholesterol	< 0.001	-0.110	0.535	0.010	0.955
VFA Flow Area	-	HDL	0.007	0.028	0.876	0.050	0.778
VFA Flow Area	-	LDL	0.006	-0.094	0.596	0.036	0.838
VFA Flow Area	-	Carotid plaque	< 0.001	0.061	0.733	0.098	0.583
Density Total	-	Density ETDRS	< 0.001	**0.735*****	< 0.001	**0.710*****	< 0.001
Density Total	-	Skeleton Total	< 0.001	-0.321	0.064	-0.211	0.230
Density Total	-	Skeleton ETDRS	< 0.001	0.293	0.093	**0.439****	0.009
Density Total	-	SBP	< 0.001	0.146	0.409	0.181	0.306
Density Total	-	DBP	< 0.001	0.139	0.433	0.184	0.297
Density Total	-	Total cholesterol	< 0.001	0.122	0.492	-0.011	0.950
Density Total	-	HDL	< 0.001	0.052	0.770	-0.106	0.551

Density Total	-	LDL	< 0.001	0.022	0.903	-0.168	0.341
Density Total	-	Carotid plaque	< 0.001	**-0.527****	0.001	**-0.509****	0.002
Density ETDRS	-	Skeleton Total	< 0.001	**-0.451****	0.007	**-0.420***	0.013
Density ETDRS	-	Skeleton ETDRS	< 0.001	**0.393***	0.022	**0.561*****	< 0.001
Density ETDRS	-	SBP	< 0.001	-0.068	0.701	-0.016	0.928
Density ETDRS	-	DBP	< 0.001	-0.325	0.060	-0.129	0.467
Density ETDRS	-	Total cholesterol	< 0.001	0.036	0.841	0.080	0.652
Density ETDRS	-	HDL	< 0.001	0.176	0.319	0.237	0.177
Density ETDRS	-	LDL	< 0.001	-0.044	0.807	-0.132	0.456
Density ETDRS	-	Carotid plaque	< 0.001	-0.223	0.205	-0.235	0.181
Skeleton Total	-	Skeleton ETDRS	0.050	-0.143	0.420	-0.166	0.349
Skeleton Total	-	SBP	< 0.001	-0.111	0.534	-0.068	0.704
Skeleton Total	-	DBP	< 0.001	0.250	0.153	0.205	0.246
Skeleton Total	-	Total cholesterol	< 0.001	**0.371***	0.031	**0.348***	0.044
Skeleton Total	-	HDL	0.002	0.158	0.373	-0.064	0.717
Skeleton Total	-	LDL	0.001	0.337	0.052	**0.396***	0.020
Skeleton Total	-	Carotid plaque	< 0.001	0.158	0.373	0.046	0.797
Skeleton ETDRS	-	SBP	0.004	0.305	0.079	0.277	0.113
Skeleton ETDRS	-	DBP	< 0.001	0.004	0.980	-0.075	0.674
Skeleton ETDRS	-	Total cholesterol	< 0.001	-0.181	0.307	-0.156	0.377
Skeleton ETDRS	-	HDL	0.003	0.245	0.162	0.166	0.347
Skeleton ETDRS	-	LDL	< 0.001	-0.263	0.132	-0.302	0.082
Skeleton ETDRS	-	Carotid plaque	< 0.001	-0.015	0.932	0.009	0.959
SBP	-	DBP	< 0.001	**0.434***	0.010	**0.420***	0.013
SBP	-	Total cholesterol	< 0.001	-0.192	0.277	-0.238	0.175
SBP	-	HDL	< 0.001	**-0.461****	0.006	**-0.479****	0.004
SBP	-	LDL	< 0.001	-0.144	0.416	-0.130	0.465
SBP	-	Carotid plaque	< 0.001	0.088	0.621	0.141	0.426
DBP	-	Total cholesterol	< 0.001	**0.374***	0.029	0.290	0.096
DBP	-	HDL	< 0.001	-0.111	0.532	-0.227	0.197
DBP	-	LDL	< 0.001	**0.362***	0.035	0.304	0.080
DBP	-	Carotid plaque	< 0.001	-0.159	0.369	-0.177	0.316
Total cholesterol	-	HDL	< 0.001	0.137	0.438	0.327	0.059
Total cholesterol	-	LDL	0.001	**0.974*****	< 0.001	**0.939*****	< 0.001
Total cholesterol	-	Carotid plaque	< 0.001	0.013	0.942	0.049	0.784
HDL	-	LDL	< 0.001	-0.005	0.980	0.140	0.430
HDL	-	Carotid plaque	< 0.001	-0.111	0.531	0.085	0.631
LDL	-	Carotid plaque	< 0.001	0.131	0.462	0.208	0.239

Table S4. Correlations among all treatment groups. * p < 0.05, ** p < .001, *** p < 0.001.

OCTA parameters, carotid Doppler ultrasound parameter and clinical values			Shapiro-Wilk	Pearson		Spearman	
			p	r	p	rho	p
NFA Area	-	FAZ Area	0.073	0.141	0.167	0.158	0.120
NFA Area	-	FAZ Perimeter	< 0.001	0.096	0.347	0.073	0.478
NFA Area	-	FAZ Circularity	0.121	0.006	0.956	-0.003	0.975
NFA Area	-	VFA Area	< 0.001	**-0.214***	0.034	-0.086	0.397
NFA Area	-	VFA Flow Area	0.337	-0.118	0.247	-0.129	0.206
NFA Area	-	Density Total	< 0.001	-0.046	0.651	0.011	0.911
NFA Area	-	Density ETDRS	< 0.001	**-0.231***	0.022	-0.128	0.208
NFA Area	-	Skeleton Total	0.081	-0.017	0.869	0.016	0.879
NFA Area	-	Skeleton ETDRS	0.055	-0.029	0.779	0.019	0.853
NFA Area	-	SBP	0.002	**0.384*****	< 0.001	**0.355*****	< 0.001
NFA Area	-	DBP	< 0.001	**0.408*****	< 0.001	**0.370*****	< 0.001
NFA Area	-	Total cholesterol	< 0.001	0.146	0.151	0.151	0.138
NFA Area	-	HDL	0.050	-0.034	0.740	0.011	0.918
NFA Area	-	LDL	< 0.001	0.118	0.248	0.107	0.296
NFA Area	-	Carotid plaque	< 0.001	**-0.242***	0.017	**-0.237***	0.019
FAZ Area	-	FAZ Perimeter	< 0.001	**0.275****	0.006	**0.257***	0.011
FAZ Area	-	FAZ Circularity	0.062	0.020	0.842	0.024	0.815
FAZ Area	-	VFA Area	< 0.001	0.037	0.721	-0.061	0.551
FAZ Area	-	VFA Flow Area	0.122	**-0.242***	0.017	**-0.273****	0.006
FAZ Area	-	Density Total	< 0.001	**-0.327****	0.001	**-0.306****	0.002
FAZ Area	-	Density ETDRS	< 0.001	**-0.294****	0.003	**-0.257***	0.011
FAZ Area	-	Skeleton Total	0.04	0.035	0.730	-0.015	0.885
FAZ Area	-	Skeleton ETDRS	0.058	-0.194	0.056	-0.181	0.074
FAZ Area	-	SBP	< 0.001	**0.305****	0.002	**0.396*****	< 0.001
FAZ Area	-	DBP	< 0.001	**0.305****	0.002	**0.327****	0.001
FAZ Area	-	Total cholesterol	< 0.001	0.157	0.122	0.113	0.268
FAZ Area	-	HDL	0.026	-0.134	0.187	-0.164	0.107
FAZ Area	-	LDL	< 0.001	0.182	0.073	0.164	0.106
FAZ Area	-	Carotid plaque	< 0.001	**0.222***	0.028	**0.214***	0.034
FAZ Perimeter	-	FAZ Circularity	< 0.001	**-0.416*****	< 0.001	**-0.452*****	< 0.001
FAZ Perimeter	-	VFA Area	< 0.001	-0.012	0.905	-0.11	0.282
FAZ Perimeter	-	VFA Flow Area	< 0.001	-0.092	0.366	-0.01	0.919
FAZ Perimeter	-	Density Total	< 0.001	-0.143	0.159	**-0.263****	0.009
FAZ Perimeter	-	Density ETDRS	< 0.001	**-0.287****	0.004	**-0.299****	0.003
FAZ Perimeter	-	Skeleton Total	< 0.001	-0.013	0.903	-0.069	0.497
FAZ Perimeter	-	Skeleton ETDRS	< 0.001	-0.192	0.059	**-0.213***	0.035
FAZ Perimeter	-	SBP	< 0.001	0.070	0.495	0.135	0.185

FAZ Perimeter	-	DBP	< 0.001	0.118	0.246	0.066	0.519
FAZ Perimeter	-	Total cholesterol	< 0.001	0.107	0.295	0.089	0.382
FAZ Perimeter	-	HDL	< 0.001	-0.062	0.544	-0.108	0.288
FAZ Perimeter	-	LDL	< 0.001	0.01	0.925	0.088	0.387
FAZ Perimeter	-	Carotid plaque	< 0.001	0.022	0.829	0.143	0.159
FAZ Circularity	-	VFA Area	< 0.001	0.191	0.06	0.084	0.413
FAZ Circularity	-	VFA Flow Area	0.358	-0.009	0.927	0.004	0.969
FAZ Circularity	-	Density Total	< 0.001	-0.012	0.905	0.07	0.491
FAZ Circularity	-	Density ETDRS	< 0.001	0.043	0.674	**0.204***	0.044
FAZ Circularity	-	Skeleton Total	0.08	-0.085	0.408	-0.063	0.537
FAZ Circularity	-	Skeleton ETDRS	0.069	0.128	0.209	0.086	0.399
FAZ Circularity	-	SBP	0.001	0.0026	0.998	0.025	0.805
FAZ Circularity	-	DBP	< 0.001	0.055	0.59	0.084	0.408
FAZ Circularity	-	Total cholesterol	< 0.001	0.170	0.094	0.116	0.256
FAZ Circularity	-	HDL	0.039	0.137	0.179	0.139	0.171
FAZ Circularity	-	LDL	< 0.001	**0.202***	0.046	0.12	0.238
FAZ Circularity	-	Carotid plaque	< 0.001	-0.158	0.12	-0.161	0.113
VFA Area	-	VFA Flow Area	< 0.001	0.019	0.851	0.053	0.601
VFA Area	-	Density Total	< 0.001	-0.099	0.332	-0.019	0.849
VFA Area	-	Density ETDRS	< 0.001	**0.212***	0.036	0.089	0.381
VFA Area	-	Skeleton Total	< 0.001	-0.003	0.977	0.081	0.429
VFA Area	-	Skeleton ETDRS	< 0.001	0.167	0.101	0.081	0.427
VFA Area	-	SBP	< 0.001	**-0.300****	0.003	-0.157	0.123
VFA Area	-	DBP	< 0.001	-0.158	0.12	-0.028	0.781
VFA Area	-	Total cholesterol	< 0.001	0.005	0.96	0.053	0.603
VFA Area	-	HDL	< 0.001	0.195	0.054	**0.240***	0.017
VFA Area	-	LDL	< 0.001	-0.059	0.561	-0.089	0.385
VFA Area	-	Carotid plaque	< 0.001	0.098	0.337	-0.044	0.665
VFA Flow Area	-	Density Total	< 0.001	-0.023	0.825	-0.005	0.958
VFA Flow Area	-	Density ETDRS	< 0.001	0.057	0.58	0.121	0.235
VFA Flow Area	-	Skeleton Total	0.259	-0.056	0.586	-0.021	0.840
VFA Flow Area	-	Skeleton ETDRS	0.22	0.085	0.405	0.137	0.179
VFA Flow Area	-	SBP	0.004	-0.183	0.071	**-0.275****	0.006
VFA Flow Area	-	DBP	< 0.001	-0.117	0.251	-0.172	0.090
VFA Flow Area	-	Total cholesterol	0.001	-0.151	0.139	-0.118	0.247
VFA Flow Area	-	HDL	0.179	0.121	0.235	0.151	0.137
VFA Flow Area	-	LDL	0.001	-0.105	0.302	-0.051	0.619
VFA Flow Area	-	Carotid plaque	< 0.001	-0.051	0.620	-0.058	0.572
Density Total	-	Density ETDRS	< 0.001	**0.686*****	< 0.001	**0.546*****	< 0.001
Density Total	-	Skeleton Total	< 0.001	-0.133	0.191	-0.076	0.460
Density Total	-	Skeleton ETDRS	< 0.001	**0.231***	0.022	**0.254***	0.012

Density Total	-	SBP	< 0.001	**-0.214***	0.034	**-0.250***	0.013
Density Total	-	DBP	< 0.001	**-0.211***	0.037	-0.195	0.054
Density Total	-	Total cholesterol	< 0.001	-0.013	0.897	-0.116	0.256
Density Total	-	HDL	< 0.001	0.172	0.091	0.174	0.086
Density Total	-	LDL	< 0.001	-0.109	0.283	**-0.225***	0.026
Density Total	-	Carotid plaque	< 0.001	**-0.274****	0.006	**-0.219***	0.030
Density ETDRS	-	Skeleton Total	< 0.001	-0.185	0.068	-0.110	0.281
Density ETDRS	-	Skeleton ETDRS	< 0.001	**0.320****	0.001	**0.351*****	< 0.001
Density ETDRS	-	SBP	< 0.001	**-0.270****	0.007	**-0.208***	0.040
Density ETDRS	-	DBP	< 0.001	**-0.306****	0.002	-0.155	0.127
Density ETDRS	-	Total cholesterol	< 0.001	-0.071	0.485	-0.063	0.536
Density ETDRS	-	HDL	< 0.001	0.194	0.056	**0.217***	0.032
Density ETDRS	-	LDL	< 0.001	-0.117	0.25	-0.104	0.306
Density ETDRS	-	Carotid plaque	< 0.001	-0.083	0.417	-0.103	0.314
Skeleton Total	-	Skeleton ETDRS	0.032	-0.066	0.515	-0.019	0.853
Skeleton Total	-	SBP	< 0.001	-0.015	0.88	0.035	0.733
Skeleton Total	-	DBP	< 0.001	0.100	0.325	0.046	0.653
Skeleton Total	-	Total cholesterol	< 0.001	0.070	0.496	0.027	0.789
Skeleton Total	-	HDL	0.020	-0.002	0.983	-0.059	0.564
Skeleton Total	-	LDL	< 0.001	-0.017	0.871	-0.057	0.580
Skeleton Total	-	Carotid plaque	< 0.001	0.196	0.054	0.145	0.153
Skeleton ETDRS	-	SBP	< 0.001	-0.061	0.552	-0.072	0.484
Skeleton ETDRS	-	DBP	< 0.001	-0.107	0.294	-0.065	0.524
Skeleton ETDRS	-	Total cholesterol	< 0.001	-0.123	0.229	-0.161	0.113
Skeleton ETDRS	-	HDL	0.054	0.159	0.118	0.137	0.178
Skeleton ETDRS	-	LDL	< 0.001	-0.035	0.730	-0.070	0.491
Skeleton ETDRS	-	Carotid plaque	< 0.001	0.020	0.842	0.018	0.857
SBP	-	DBP	< 0.001	**0.690*****	< 0.001	**0.766*****	< 0.001
SBP	-	Total cholesterol	< 0.001	0.183	0.071	**0.201***	0.047
SBP	-	HDL	< 0.001	**-0.434*****	< 0.001	**-0.387*****	< 0.001
SBP	-	LDL	< 0.001	**0.206***	0.041	**0.213***	0.036
SBP	-	Carotid plaque	< 0.001	0.023	0.825	-0.013	0.899
DBP	-	Total cholesterol	< 0.001	**0.314****	0.002	**0.260****	0.010
DBP	-	HDL	< 0.001	**-0.313****	0.002	**-0.242***	0.016
DBP	-	LDL	< 0.001	**0.323****	0.001	**0.288****	0.004
DBP	-	Carotid plaque	< 0.001	-0.027	0.792	-0.063	0.537
Total cholesterol	-	HDL	< 0.001	0.197	0.051	**0.278****	0.006
Total cholesterol	-	LDL	< 0.001	**0.888*****	< 0.001	**0.836*****	< 0.001
Total cholesterol	-	Carotid plaque	< 0.001	0.018	0.861	-0.0007	0.994
HDL	-	LDL	< 0.001	0.099	0.334	0.143	0.160

HDL	-	Carotid plaque	< 0.001	-0.122	0.232	-0.121	0.234
LDL	-	Carotid plaque	< 0.001	0.097	0.344	0.110	0.281

Correlations among demographic parameters and ultrasound were calculated and are displayed in Table S5. Area did not prove to be relevant.

Table S5. Demographic and ultrasound parameters. *p < 0.05, **p < 0.01, ***p < 0.001. PSV: peak systolic velocity; EDV: end-diastolic velocity; ICA: internal carotid artery; ECA: external carotid artery; CCA: common carotid artery; VA: vertebral artery; IR: Resistive Index; BMI: body mass index.

Demographic Parameters		Ultrasound Parameters	Pearson		Spearman	
			r	p	rho	p
Age	-	ICA EDV	**0.257***	0.011	0.149	0.143
Age	-	CCA IR	**0.253***	0.012	**0.264****	0.009
BMI	-	ICA IR	-0.126	0.215	**-0.212***	0.036
BMI	-	ECA IR	-0.197	0.052	**-0.210***	0.037
BMI	-	CCA IR	**-0.282****	0.005	**-0.238***	0.018
BMI	-	VA EDV	**0.250***	0.013	**0.247***	0.014
BMI	-	VA IR	**-0.354*****	< 0.001	**-0.405*****	< 0.001
Smokers	-	ECA PSV	-0.177	0.081	**-0.201***	0.048

Shapiro–Wilk test is calculated in Table S6.

Table S6. Shapiro–Wilk test. OCTA: optical coherence tomography angiography; FAZ: foveal avascular zone; NFA: non-flow area; VFA: vascular flow area; ETDRS: Early Treatment of Diabetic Retinopathy Study; ACEI: Angiotensin-converting-enzyme inhibitors; CCB: Calcium channel blockers; ARB: Angiotensin Receptor Blocker.

**OCTA parameters**	**Medication group**		
	**ACEI+statins (N= 34)**	**CCB+statins (N= 30)**	**ACEI/ARB+statins (N = 34)**
	**p-value**	**p-value**	**p-value**
NFA Area	0.048*	0.780	0.130
FAZ Area	0.008*	0.064	0.165
FAZ Perimeter	< 0.001*	< 0.001*	< 0.001*
FAZ Circularity	0.490	0.703	0.176

VFA Area	0.003*	0.028*	< 0.001*
VFA Flow Area	0.426	0.966	0.571
Density Total	0.377	0.892	< 0.001*
Density ETDRS	0.298	0.061	< 0.001*
Skeleton Total	0.073	0.236	0.016*
Skeleton ETDRS	0.564	0.024*	0.436

Brown-Forsyth - Robust Tests of Equality of Means is displayed in Table S7. We used the Brown-Forsythe test as a robust statistical method in order to check for equality of variances across the three groups.

Table S7. Brown-Forsyth - Robust Tests of Equality of Means. Asymptotically F distributed. b. Statistically significant OCTA parameters. p<0.05. OCTA: optical coherence tomography angiography; FAZ: foveal avascular zone; NFA: non-flow area; VFA: vascular flow area; ETDRS: Early Treatment of Diabetic Retinopathy Study.

OCTA parameters	Statistic[a]	Sig.
NFA Area[b]	14.166	**< 0.001**
FAZ Area[b]	18.049	**< 0.001**
FAZ Perimeter	0.093	0.911
FAZ Circularity[b]	4.042	**0.021**
VFA Area	2.096	0.138
VFA Flow Area[b]	3.316	**0.041**
Density Total[b]	6.823	**0.002**
Density ETDRS	6.217	**0.004**
Skeleton Total[b]	2.244	0.113
Skeleton ETDRS	2.281	0.108

Table S8. shows the Kruskal Wallis Test.

Table S8. Kruskal Wallis Test. OCTA: optical coherence tomography angiography; FAZ: foveal avascular zone; NFA: non-flow area; VFA: vascular flow area; ETDRS: Early Treatment of Diabetic Retinopathy Study; ACEI: Angiotensin-converting-enzyme inhibitors; CCB: Calcium channel blockers; ARB: Angiotensin Receptor Blocker.

	Medication groups

OCTA parameters	ACEI+statins (N= 34) Mean Rank	CCB+statins (N= 30) Mean Rank	ACEI/ARB+statins (N = 34) Mean Rank	*KRUSKAL-WALLIS* *p*-value
NFA Area*	35.22	48.12	65.00	**< 0.001**
FAZ Area*	28.79	57.27	63.35	**< 0.001**
FAZ Perimeter	47.19	47.17	53.87	0.541
FAZ Circularity*	53.90	38.12	55.15	**0.031**
VFA Area	52.24	53.10	43.59	0.307
VFA Flow Area*	58.46	51.28	38.97	**0.017**
Density Total*	59.96	45.78	42.32	**0.026**
Density ETDRS*	59.15	46.03	42.91	**0.045**
Skeleton Total	46.79	58.10	44.62	0.131
Skeleton ETDRS	48.68	57.70	43.09	0.119

Table S9**.** Dunn's Post Hoc Comparisons are displayed.

Table S9. Dunn's Post Hoc Comparisons. OCTA: optical coherence tomography angiography; FAZ: foveal avascular zone; NFA: non-flow area; VFA: vascular flow area; ETDRS: Early Treatment of Diabetic Retinopathy Study; ACEI: Angiotensin-converting-enzyme inhibitors; CCB: Calcium channel blockers; ARB: Angiotensin Receptor Blocker.

OCTA parameters	Multiple comparisons		p-value
NFA Area	ACEI/ARB+statins	ACEI+statins	**< 0.001**
		CCB+statins	**0.035**
FAZ Area	ACEI+statins	CCB+statins	**< 0.001**
		ACEI/ARB+statins	**< 0.001**
FAZ Circularity	CCB+statins	ACEI+statins	0.053
		ACEI/ARB+statins	**0.050**
VFA Flow Area	ACEI+statins	CCB+statins	0.314
		ACEI/ARB+statins	**0.014**
Density Total	ACEI+statins	CCB+statins	0.093
		ACEI/ARB+statins	**0.032**

Density ETDRS	ACEI+statins	CCB+statins	0.065
		ACEI/ARB+statins	**0.019**

Spearman and Pearson correlations were used among OCTA parameters, carotid Doppler ultrasound and clinical measurements. Table S10. displayes the test results.

Table S10. All therapy lots. Pearson and Spearman correlations. * p < 0.05, ** p < 0.01, *** p < 0.001. FAZ: foveal avascular zone; NFA: non-flow area; VFA: vascular flow area; ETDRS: Early Treatment of Diabetic Retinopathy Study; SBP: systolic blood pressure; DBP: diastolic blood pressure; HDL: high-density lipoprotein; LDL: low-density lipoprotein.

**OCTA parameters and clinical values**			**Shapiro-Wilk**	**Pearson**		**Spearman**	
			**p**	**r**	**p**	**rho**	**p**
NFA Area	-	VFA Area	< 0.001	-0.214*	0.034	-0.086	0.397
NFA Area	-	Density ETDRS	< 0.001	-0.231*	0.022	-0.128	0.208
NFA Area	-	SBP	0.002	0.384***	< 0.001	0.355***	< 0.001
NFA Area	-	DBP	< 0.001	0.408***	< 0.001	0.370***	< 0.001
NFA Area	-	Carotid plaque	< 0.001	-0.242*	0.017	-0.237*	0.019
FAZ Area	-	FAZ Perimeter	< 0.001	0.275**	0.006	0.257*	0.011
FAZ Area	-	VFA Flow Area	0.122	-0.242*	0.017	-0.273**	0.006
FAZ Area	-	Density Total	< 0.001	-0.327**	0.001	-0.306**	0.002
FAZ Area	-	Density ETDRS	< 0.001	-0.294**	0.003	-0.257*	0.011
FAZ Area	-	SBP	< 0.001	0.305**	0.002	0.396***	< 0.001
FAZ Area	-	DBP	< 0.001	0.305**	0.002	0.327**	0.001
FAZ Area	-	Carotid plaque	< 0.001	0.222*	0.028	0.214*	0.034
FAZ Perimeter	-	FAZ Circularity	< 0.001	-0.416***	< 0.001	-0.452***	< 0.001
FAZ Perimeter	-	Density Total	< 0.001	-0.143	0.159	-0.263**	0.009
FAZ Perimeter	-	Density ETDRS	< 0.001	-0.287**	0.004	-0.299**	0.003
FAZ Perimeter	-	Skeleton ETDRS	< 0.001	-0.192	0.059	-0.213*	0.035

FAZ Circularity	-	Density ETDRS	< 0.001	0.043	0.674	0.204*	0.044
FAZ Circularity	-	LDL	< 0.001	0.202*	0.046	0.12	0.238
VFA Area	-	Density ETDRS	< 0.001	0.212*	0.036	0.089	0.381
VFA Area	-	SBP	< 0.001	-0.300**	0.003	-0.157	0.123
VFA Area	-	HDL	< 0.001	0.195	0.054	0.240*	0.017
VFA Flow Area	-	SBP	0.004	-0.183	0.071	-0.275**	0.006
Density Total	-	Density ETDRS	< 0.001	0.686***	< 0.001	0.546***	< 0.001
Density Total	-	Skeleton ETDRS	< 0.001	0.231*	0.022	0.254*	0.012
Density Total	-	SBP	< 0.001	-0.214*	0.034	-0.250*	0.013
Density Total	-	DBP	< 0.001	-0.211*	0.037	-0.195	0.054
Density Total	-	LDL	< 0.001	-0.109	0.283	-0.225*	0.026
Density Total	-	Carotid plaque	< 0.001	-0.274**	0.006	-0.219*	0.030
Density ETDRS	-	Skeleton ETDRS	< 0.001	0.320**	0.001	0.351***	< 0.001
Density ETDRS	-	SBP	< 0.001	-0.270**	0.007	-0.208*	0.040
Density ETDRS	-	DBP	< 0.001	-0.306**	0.002	-0.155	0.127
Density ETDRS	-	HDL	< 0.001	0.194	0.056	0.217*	0.032
SBP	-	DBP	< 0.001	0.690***	< 0.001	0.766***	< 0.001
SBP	-	Total cholesterol	< 0.001	0.183	0.071	0.201*	0.047
SBP	-	HDL	< 0.001	-0.434***	< 0.001	-0.387***	< 0.001
SBP	-	LDL	< 0.001	0.206*	0.041	0.213*	0.036
DBP	-	Total cholesterol	< 0.001	0.314**	0.002	0.260**	0.010
DBP	-	HDL	< 0.001	-0.313**	0.002	-0.242*	0.016

Tabel S11. Estimated Marginal Means & Pairwise Comparisons.

Dependent Variable	Medication groups	Mean	Std. Error	95% Confidence Interval	
				Lower Bound	Upper Bound
NFA Area	ACEI+statins	0.356[a]	0.028	0.301	0.411

	CCB+statins	0.424[a]	0.023	0.378	0.470
	ACEI/ARB+statins	0.504[a]	0.030	0.444	0.564
FAZ Area	ACEI+statins	0.366[a]	0.042	0.284	0.449
	CCB+statins	0.602[a]	0.035	0.533	0.671
	ACEI/ARB+statins	0.622[a]	0.045	0.532	0.711
FAZ Perimeter	ACEI+statins	3.374[a]	0.484	2.412	4.336
	CCB+statins	3.673[a]	0.404	2.869	4.476
	ACEI/ARB+statins	0.498[a]	0.036	0.427	0.568
FAZ Circularity	ACEI+statins	0.514[a]	0.033	0.449	0.579
	CCB+statins	0.417[a]	0.027	0.363	0.472
	ACEI/ARB+statins	3.583[a]	0.526	2.537	4.629
VFA Area	ACEI+statins	3.128[a]	0.016	3.097	3.160
	CCB+statins	3.149[a]	0.013	3.123	3.175
	ACEI/ARB+statins	3.149[a]	0.017	3.115	3.183
VFA Flow Area	ACEI+statins	1.479[a]	0.071	1.338	1.619
	CCB+statins	1.399[a]	0.059	1.282	1.516
	ACEI/ARB+statins	1.172[a]	0.077	1.020	1.325
Density Total	ACEI+statins	36.148[a]	0.747	34.663	37.634
	CCB+statins	34.802[a]	0.624	33.561	36.043
	ACEI/ARB+statins	33.370[a]	0.813	31.755	34.985
Density ETDRS	ACEI+statins	35.385[a]	0.857	33.683	37.088
	CCB+statins	33.693[a]	0.716	32.271	35.115
	ACEI/ARB+statins	31.953[a]	0.932	30.102	33.804
Schleton Total	ACEI+statins	20.555[a]	0.245	20.068	21.041
	CCB+statins	21.177[a]	0.205	20.771	21.583
	ACEI/ARB+statins	20.745[a]	0.266	20.216	21.274
Scheleton ETDRS	ACEI+statins	21.087[a]	0.301	20.488	21.686
	CCB+statins	21.189[a]	0.252	20.689	21.690

	ACEI/ARB+statins	20.108[a]	0.328	19.457	20.759
